# Hypothalamic inhibition of socio-sexual behaviour by increasing neuroestrogen synthesis

**DOI:** 10.1038/ncomms4061

**Published:** 2014-01-16

**Authors:** Takayoshi Ubuka, Shogo Haraguchi, Yasuko Tobari, Misato Narihiro, Kei Ishikawa, Takanori Hayashi, Nobuhiro Harada, Kazuyoshi Tsutsui

**Affiliations:** 1 Department of Biology, Waseda University, 2-2 Wakamatsu-cho, Shinjuku, Tokyo 162-8480, Japan; 2 Department of Biochemistry, Fujita Health University School of Medicine, Toyoake, Aichi 470-1192, Japan

## Abstract

Gonadotropin-inhibitory hormone (GnIH) is a hypothalamic neuropeptide that inhibits gonadotropin secretion and socio-sexual behaviours. Oestrogen (neuroestrogen) synthesized in the brain from androgen by aromatase regulates male socio-sexual behaviours. Here we show that GnIH directly activates aromatase and increases neuroestrogen synthesis in the preoptic area (POA) and inhibits socio-sexual behaviours of male quail. Aromatase activity and neuroestrogen concentration in the POA are low in the morning when the birds are active, but neuroestrogen synthesis gradually increases until the evening when the birds become inactive. Centrally administered GnIH in the morning increases neuroestrogen synthesis in the POA and decreases socio-sexual behaviours. Centrally administered 17β-oestradiol at higher doses also inhibits socio-sexual behaviours in the morning. These results suggest that GnIH inhibits male socio-sexual behaviours by increasing neuroestrogen synthesis beyond its optimum concentration for the expression of socio-sexual behaviours. This is the first demonstration of any hypothalamic neuropeptide that directly regulates neuroestrogen synthesis.

## Abstract (web summary)

The conversion of testosterone into oestrogen in the brain is implicated in male aggressive behaviour. Ubuka *et al.* show that gonadotropin-inhibitory hormone inhibits male aggression by increasing oestrogen synthesis in the brain beyond its optimum concentration for the expression of aggressive behaviour.

## Graphical abstract

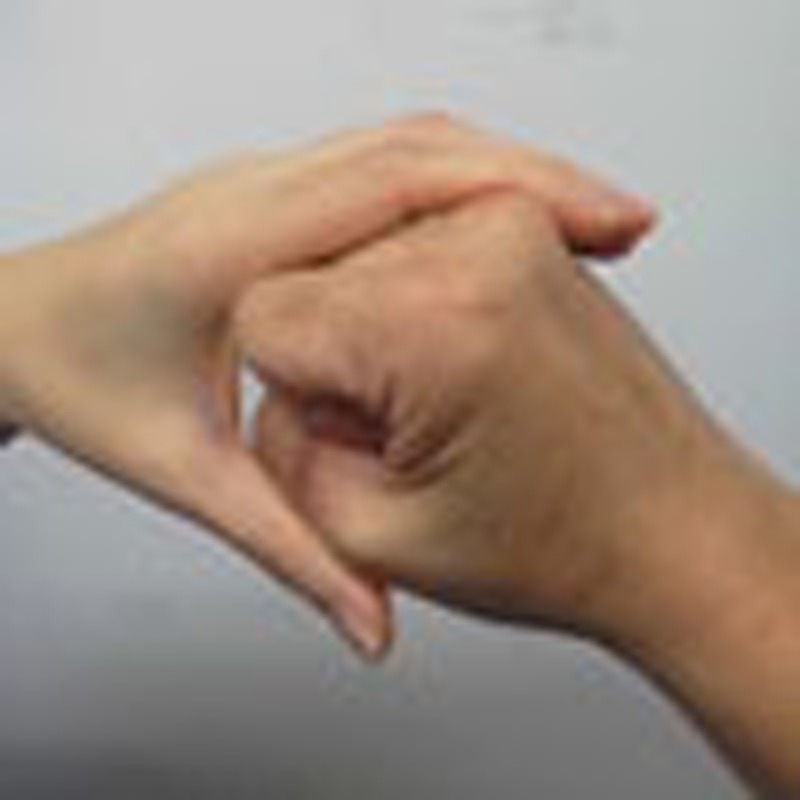

Gonadotropin-inhibitory hormone (GnIH) is a hypothalamic neuropeptide that inhibits gonadotropin secretion from the pituitary, which was first demonstrated in the Japanese quail (*Coturnix japonica*)^1^,^2^,^3^,^4^,^5^,^6^. GnIH is synthesized in neurons of the paraventricular nucleus (PVN) in birds^1^,^7^,^8^. GnIH neurons not only project to the median eminence to control anterior pituitary hormone secretion^1^,^7^,^8^ but also to other brain areas, such as the preoptic area (POA)^9^ and the periaqueductal central grey (PAG)^9^, suggesting that GnIH directly regulates socio-sexual behaviours, such as aggressive and sexual behaviours^10^,^11^. The mRNA of the cognate G-protein-coupled receptor (GPR147) for GnIH is also expressed in the POA and PAG^9^,^12^. It was shown that central administration of GnIH to the third ventricle of female white-crowned sparrows (*Zonotrichia leucophrys gambelii*) inhibited copulation solicitation, which is analogous to mammalian lordosis^13^.

To understand the function of GnIH neurons in the brain, we previously investigated the effect of RNA interference (RNAi) of the *GnIH* gene on the behaviour of white-crowned sparrows, a highly social songbird species. Administration of small interfering RNA (siRNA) against GnIH precursor mRNA into the third ventricle of male and female birds increased locomotor activity and stimulated agonistic vocalizations^14^. GnIH RNAi further enhanced territorial song of male birds when they were challenged by novel male songs^14^. The overall results suggested that *GnIH* gene silencing induces socio-sexual arousal^14^. It was thus hypothesized that GnIH decreases socio-sexual arousal resulting in the inhibition of socio-sexual behaviours^14^,^15^,^16^.

Japanese quail is a commonly used laboratory species for studies of socio-sexual behaviours and its neurophysiological and neuroendocrine bases^17^. Sexually mature male quail frequently fight with intense aggressiveness, displaying a series of stereotyped actions^17^,^18^. Aggressive behaviour of male quail is considered to be androgen dependent because it is reduced by castration and restored by androgen treatment^17^,^18^. However, there is generally no correlation between the order of aggressiveness of adult male quail and peripheral testosterone concentration^19^,^20^. It is considered that full expression of testosterone action in the brain requires its aromatization to 17β-oestradiol (E2) because aggression of inactive males is only activated by aromatizable androgen (testosterone and androstenedione) or E2, but not by non-aromatizable androgen (dihydrotestosterone), and testosterone-induced aggression is blocked by co-administration of aromatase inhibitors^20^,^21^.

Although it is generally thought that the action of testosterone on male socio-sexual behaviours requires its aromatization into oestrogen (neuroestrogen) in the brain (the aromatization hypothesis)^22^,^23^,^24^, the precise mechanism remains unclear. We initially hypothesized that GnIH may inhibit socio-sexual behaviours of male quail by inhibiting aromatase activity (AA) and neuroestrogen synthesis in the brain. Before investigating the effect of GnIH administration, we first measured daily changes in socio-sexual activities of male quail. We find that socio-sexual activities of male birds are high in the morning and gradually decrease until the evening. Accordingly, we centrally administer GnIH in the morning and show that GnIH decreases socio-sexual activities of male birds. We also investigate the effect of GnIH RNAi and concomitant central administration of GnIH on socio-sexual activities of male birds. The POA is thought to be the critical site of aromatization and neuroestrogen action for the activation of copulatory behaviour of male quail^25^,^26^. Accordingly, we investigate whether GnIH neuronal fibres innervate aromatase cells and also whether aromatase cells express GnIH receptor mRNA in the POA. We next measure daily changes in GnIH content and release, GPR147 mRNA expression, AA, E2 content and release in the brain blocks including the POA. GnIH is actively released throughout the day in the POA, and AA and E2 content and release in the POA are low in the morning and gradually increased until the evening. Because socio-sexual activities of male quail were high in the morning and gradually decrease until the evening, we thought GnIH may gradually activate aromatase and increase E2 synthesis from the morning until the evening in the POA, and higher concentration of neuroestrogen may inhibit male socio-sexual behaviours in the evening. Accordingly, we investigate the effect of GnIH administration on AA and E2 synthesis in the POA *in vitro* and *in vivo*. GnIH stimulated the activity of aromatase and increased E2 synthesis in the POA *in vitro* and *in vivo*. Finally, we test the effect of central administration of E2 on socio-sexual behaviours of male quail and show that higher doses of E2 inhibit male socio-sexual behaviours. The overall results suggest that GnIH directly activates aromatase and increases neuroestrogen synthesis in the POA and inhibits the expression of socio-sexual behaviours of male quail.

## Results

### Daily change in socio-sexual activities of male quail

When sexually active male quail are paired in a relatively small cage they display sequential stereotypic actions. They often threaten the opponent by stretching the neck and walking around (Struts), approach and chase, peck the opponent (Pecks), grab the back of the opponent’s head or neck with their beak (Grabs), attempt to ride on the back of the opponent (Mounts), ride on the back of the opponent and lower their cloaca close to the opponent’s cloaca (Cloacal contact (CC)-like actions) (Supplementary Fig. 1). The frequency of these actions represents the activity of socio-sexual behaviours of male quail^17^,^18^,^19^,^20^,^21^.

**Figure 1:**
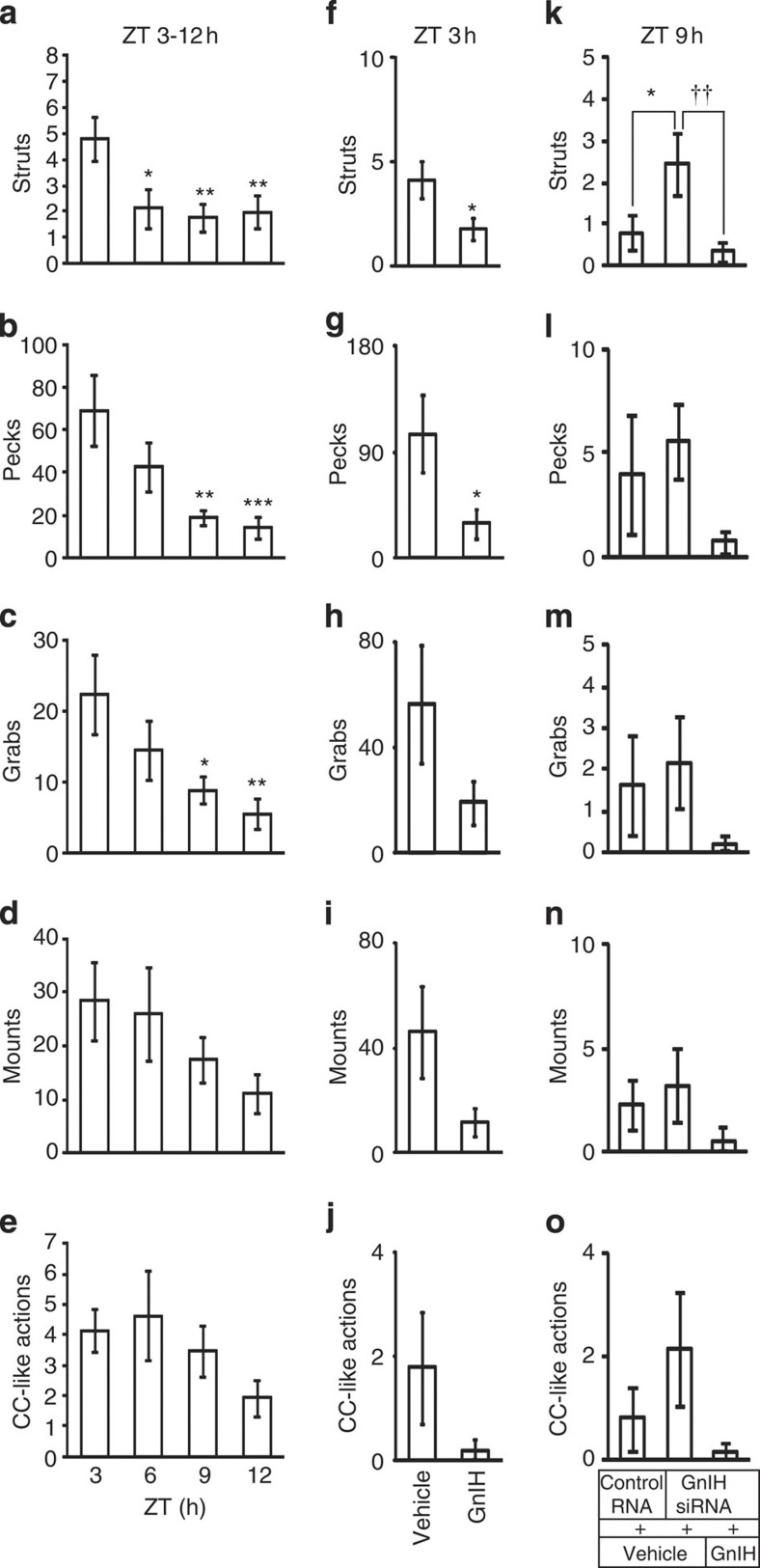
Diurnal changes and the effects of GnIH and/or GnIH RNAi on socio-sexual actions of male quail. Number of Struts (**a**,**f**,**k**), Pecks (**b**,**g**,**l**), Grabs (**c**,**h**,**m**), Mounts (**d**,**i**,**n**), and Cloacal contact-like (CC-like) actions (**e**,**j**,**o**) was counted in 5 min. (**a**–**e**) Diurnal socio-sexual behaviours were recorded during ZT 2.5 and 3.5 h (ZT 3), ZT 5.5 and 6.5 h (ZT 6), ZT 8.5 and 9.5 h (ZT 9), and ZT 11.5 and 12.5 h (ZT 12). The columns and the vertical lines represent the mean±s.e.m. (*n*=8). (**a**) Degrees of freedom (DOF)=31, F=4.2, *P*=0.015 by one-way ANOVA; *P<0.05, **P<0.01 versus ZT 3 by Fisher’s PLSD. (**b**) DOF=31, F=5.8, *P*=0.0032 by one-way ANOVA; **P<0.01, ***P<0.001 versus ZT 3 by Fisher’s PLSD. (**c**) DOF=31, F=3.8, *P*=0.020 by one-way ANOVA; *P<0.05, **P<0.01 versus ZT 3 by Fisher’s PLSD. (**f**–**j**) Effect of central administration of GnIH on socio-sexual activities of male quail. The columns and the vertical lines represent the mean±s.e.m. (*n*=12). (**f**) DOF=22, *t*=2.2, *P=0.041 by two-tailed Student’s *t*-test. (**g**) DOF=22, *t*=2.1, *P=0.045 by two-tailed Student’s *t*-test. (**k**–**o**) Effect of GnIH RNAi and GnIH administration on socio-sexual activities of male quail. The columns and the vertical lines represent the mean±s.e.m. (*n*=8). (**k**) DOF=23, F=4.5, *P*=0.024 by one-way ANOVA; *P<0.05, ^††^*P*<0.01 by Fisher’s PLSD.

We first measured daily changes in socio-sexual activities of sexually active adult male quail. All quail were kept under long day photoperiods (16 h light, 8 h dark) to keep them sexually active. We counted the number of Struts, Pecks, Grabs, Mounts and CC-like actions in 5 min during the light hours around zietgeiber time (ZT) 3, 6, 9 and 12 h. The frequency of Struts, Pecks and Grabs was significantly high in the morning (ZT 3 h) and decreased in the afternoon (ZT 9 h) and the evening (ZT 12 h) (Fig. 1a–c). The frequency of Mounts and CC-like actions tended to decrease towards the evening (Fig. 1d,e).

### Inhibition of socio-sexual activities by GnIH

We then tested the effect of central administration of GnIH on socio-sexual activities of male quail in the morning (ZT 3 h) when their natural expression is high (Fig. 1a–e). Intracerebroventricular (ICV) administration of GnIH inhibited the number of Struts (Fig. 1f) and Pecks (Fig. 1g) in 5 min, quantified 30 min after administration. Similar trends in the number of Grabs (Fig. 1h), Mounts (Fig. 1i) and CC-like actions (Fig. 1j) by GnIH administration were observed. To investigate the relationships between the frequencies of socio-sexual activities and peripheral testosterone concentration, we took blood samples immediately after the behavioural tests and measured testosterone concentration in the serum. Central administration of GnIH did not decrease serum testosterone concentration 30 min after administration (Supplementary Fig. 2a). There was no correlation between testosterone concentration in the serum and the number of Struts (*n*=24, R=0.24, *P*=0.26 by two-sided Pearson’s correlation test; Supplementary Fig. 2b), Pecks (*n*=24, R=0.07, *P*=0.75 by two-sided Pearson’s correlation test; Supplementary Fig. 2c), Grabs (*n*=24, R=0.14, *P*=0.52 by two-sided Pearson’s correlation test), Mounts (*n*=24, R=0.15, *P*=0.48 by two-sided Pearson’s correlation test) or CC-like actions (*n*=24, R=−0.11, *P*=0.62 by two-sided Pearson’s correlation test) in all birds tested.

We also tested the effect of *GnIH* gene silencing and central administration of GnIH on socio-sexual behaviours of male quail. The number of five stereotypic actions of socio-sexual behaviours was compared among the birds that were administered with control RNA or GnIH siRNA the day before the behavioural test because 1 day treatment of GnIH siRNA most effectively suppressed the expression of GnIH mRNA in the PVN (Supplementary Fig. 3a) and GnIH content in the POA (Supplementary Fig. 3b). The number of socio-sexual activities was compared in the evening when their natural expression was low (Fig. 1a–e) among the birds that were administered with control RNA the day before and administered with vehicle 35 min before the behavioural test (Control RNA+vehicle), the birds that were administered with GnIH siRNA the day before and administered with vehicle 35 min before the behavioural test (GnIH siRNA+vehicle), and the birds that were administered with GnIH siRNA the day before and administered with GnIH 35 min before the behavioural test (GnIH siRNA+GnIH) (Supplementary Fig. 3c). GnIH RNAi significantly increased the number of Struts (Fig. 1k). In contrast, central administration of GnIH decreased the number of Struts induced by GnIH RNAi treatment (Fig. 1k). The number of Pecks, Grabs, Mounts and CC-like actions showed similar trends by GnIH RNAi and GnIH administration (Fig. 1l–o).

**Figure 2:**
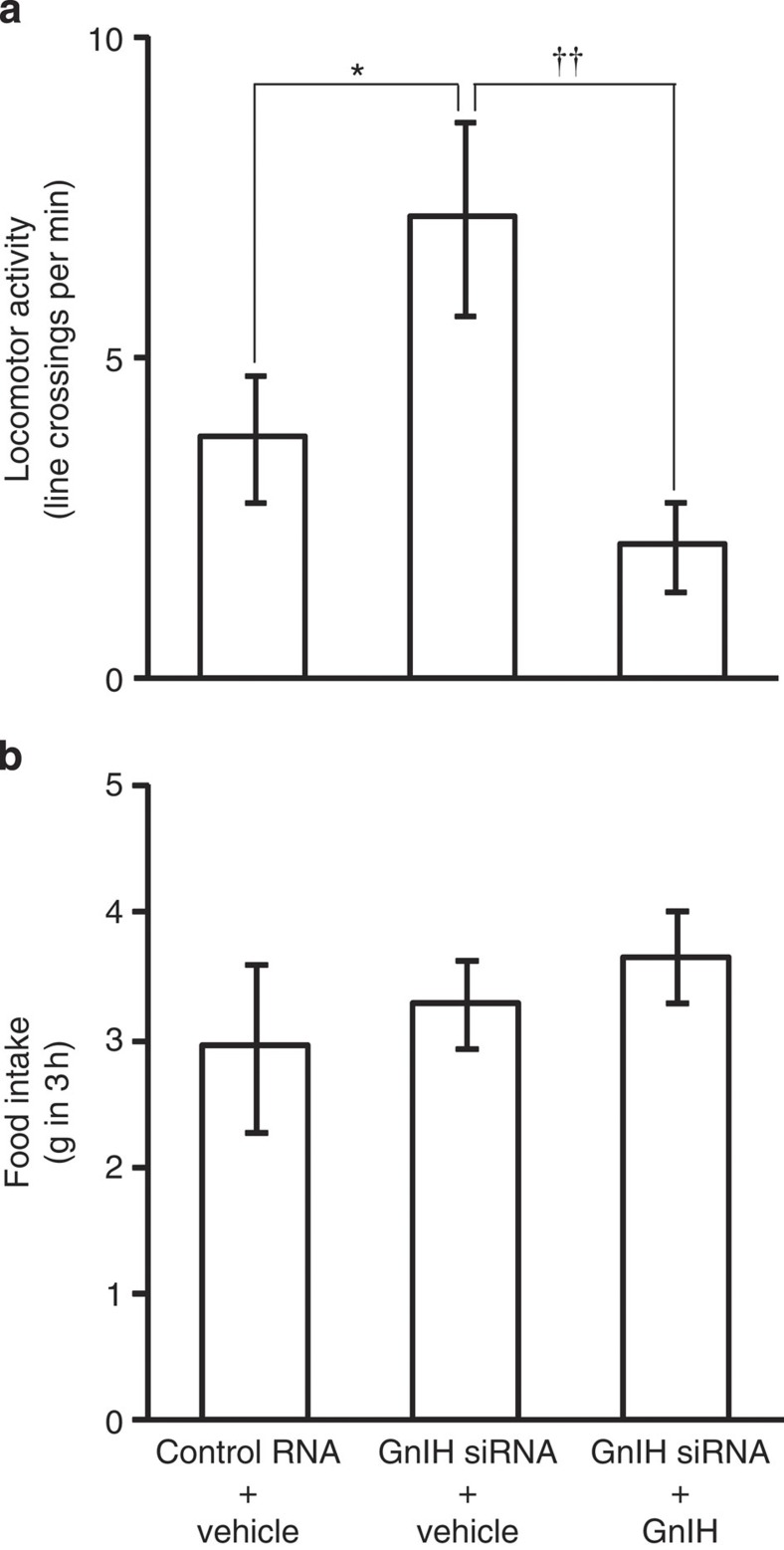
Effect of GnIH RNAi and central administration of GnIH on locomotor activity and food intake. (**a**) Effect of GnIH RNAi and central administration of GnIH on locomotor activity. The columns and the vertical lines represent the mean ±s.e.m. (*n*=8). DOF=23, F=5.2, *P*=0.015 by one-way ANOVA; *P<0.05, ^††^*P*<0.01 by Fisher’s PLSD. (**b**) Effects of GnIH RNAi and central administration of GnIH on food intake. The columns and the vertical lines represent the mean±s.e.m. (*n*=12).

Locomotor activity was also compared among the birds that were administered with control RNA the day before and administered with vehicle 30 min before the behavioural test (Control RNA+Vehicle), the birds that were administered with GnIH siRNA the day before and administered with vehicle 30 min before the behavioural test (GnIH siRNA+vehicle), and the birds that were administered with GnIH siRNA the day before and administered with GnIH 30 min before the behavioural test (GnIH siRNA+GnIH) as a measurement of socio-sexual arousal^14^ (Supplementary Fig. 3c). GnIH RNAi significantly increased locomotor activity (Fig. 2a) and central administration of GnIH decreased locomotor activity induced by GnIH RNAi treatment (Fig. 2a). On the contrary, no effect of GnIH RNAi or GnIH administration was observed on food intake in 3 h that was measured immediately after the behavioural tests (Fig. 2b) (Supplementary Fig. 3c).

### GnIH neuronal fibres project to aromatase cells

**Figure 3:**
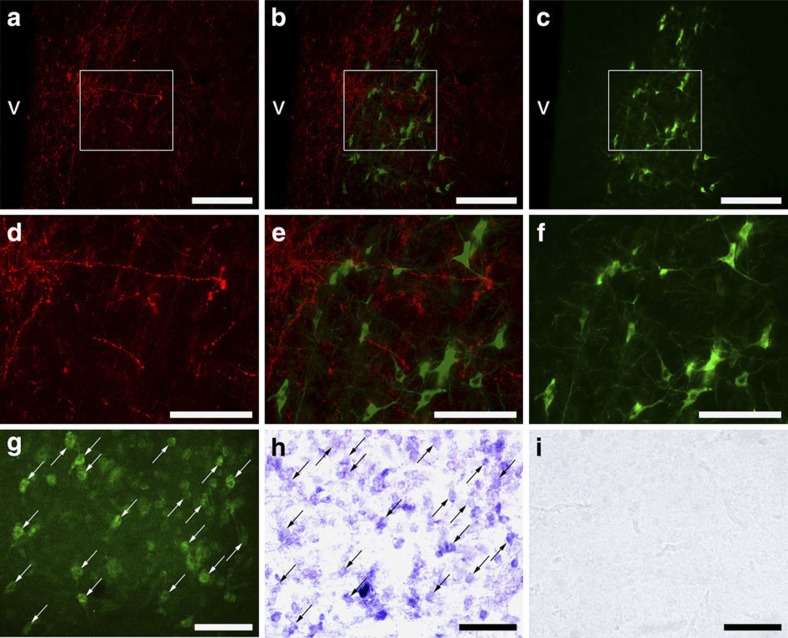
Distribution of GnIH-ir fibres and aromatase-ir cells and GnIH receptor mRNA in the POA. (**a**) Abundant GnIH-ir neuronal fibres were observed in the POA. v, third ventricle. Bar, 100 μm. (**c**) A cluster of aromatase-ir cells was observed in the POA. v, third ventricle. Bar, 100 μm. (**b**) The merged image of the pictures (**a**) and (**c**) showed abundant GnIH-ir neuronal fibres in the vicinity of aromatase-ir cells. v, third ventricle. Bar, 100 μm. Similar results were obtained in repeated experiments using four different birds. (**d**–**f**) Higher magnification of the blocked area in (**a**–**c**). Bar, 50 μm. (**g**–**i**) Aromatase immunohistochemistry (**g**) and *in situ* hybridization for GnIH receptor (GPR147) mRNA on the same sections showed that almost all aromatase-ir cells express GPR147 mRNA. Arrows in (**g**) and (**h**) indicate identical cells in the POA. Bar, 50 μm. *In situ* hybridization using sense RNA probe (**i**) served as controls. Similar results were obtained in repeated experiments using three different birds.

The behavioural tests and measurement of testosterone concentration in the sera sampled immediately after the behavioural tests indicated that GnIH inhibits socio-sexual behaviour of male quail independent of peripheral testosterone concentration. Previous studies have suggested that full expression of testosterone action in the brain requires its aromatization^19^,^20^,^21^,^22^,^23^,^24^,^25^,^26^. GnIH may inhibit socio-sexual behaviour of male quail by regulating neuroestrogen synthesis. We therefore investigated the relative location of GnIH-ir neuronal fibres and aromatase-ir cells. Abundant GnIH-ir neuronal fibres (Fig. 3a,b,d,e) and aromatase-ir cells (Fig. 3b,c,e–g) were observed in the POA. Merged image of GnIH-ir neuronal fibres and aromatase-ir cells showed close appositions of GnIH-ir neuronal fibres in the vicinity of aromatase-ir cells in the POA (Fig. 3b,e). Abundant GnIH-ir neuronal fibres and aromatase-ir cells were not only observed in the POA (Supplementary Fig. 4a) but also in the bed nucleus of the stria terminalis (BSTM) (Supplementary Fig. 4b), mediobasal hypothalamus (MBH) (Supplementary Fig. 4c) and PAG (Supplementary Fig. 4d), where aromatase mRNA is distinctively expressed in the quail brain^27^. Close appositions of GnIH-ir neuronal fibres were also observed in the vicinity of aromatase-ir cells in these brain regions (Supplementary Fig. 4a–d).

### Aromatase cells express GnIH receptor mRNA

To investigate if GnIH may directly regulate aromatase cells, we further analysed the expression of GnIH receptor (GPR147) mRNA in aromatase-ir cells by *in situ* hybridization for GPR147 mRNA combined with aromatase immunohistochemistry in the POA. Almost all aromatase-ir cells observed expressed GPR147 mRNA in the POA (Fig. 3g,h). Together with the results of GnIH-ir fibres observed in the vicinity of aromatase-ir cells (Fig. 3b,e), it is highly possible that GnIH directly regulates aromatase cells in the POA.

### Daily changes in GnIH and AA and E2 content in the brain

**Figure 4:**
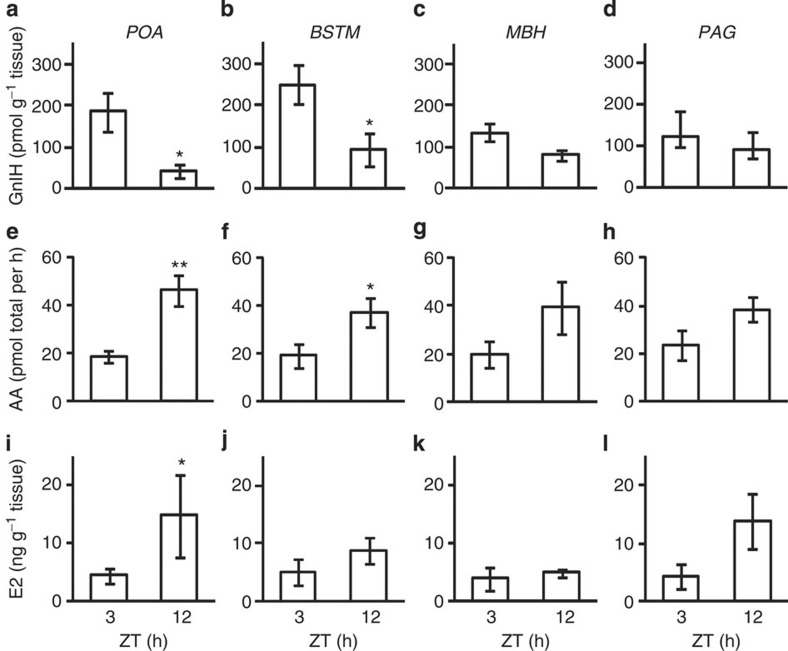
Diurnal changes in GnIH content and AA and E2 content in the quail brain. The quail brains were collected between ZT 2.5 and 3.5 h (ZT 3) or ZT 11.5 and 12.5 h (ZT 12). The brain regions including preoptic area (POA), bed nucleus of the stria terminalis (BSTM), mediobasal hypothalamus (MBH) and periaqueductal grey (PAG) were separated according to the regions described in Supplementary Fig. 5. (**a**–**d**) Diurnal changes in GnIH concentration in the quail brain. The columns and the vertical lines represent the mean±s.e.m. (*n*=5). (**a**), DOF=8, *t*=2.9, *P=0.021 by two-tailed Student’s *t*-test. (**b**) DOF=8, *t*=2.5, *P=0.036 by two-tailed Student’s *t*-test. (**e**–**h**) Diurnal changes in AA in the quail brain. The columns and the vertical lines represent the mean±s.e.m. (*n*=5). (**e**) DOF=8, *t*=−4.1, **P=0.0036 by two-tailed Student’s *t*-test. (**f**) DOF=8, *t*=−2.4, *P=0.045 by two-tailed Student’s *t*-test. (**i**–**l**) Diurnal changes in E2 concentration in the quail brain. The columns and the vertical lines represent the mean±s.e.m. (*n*=5). (**i**) U=3, Z=−2.0, *P=0.047 by Mann–Whitney *U-*test.

To measure daily changes in GnIH content, AA and E2 content in distinct brain regions, quail brains were sampled in the morning (ZT 3 h) when the quail are active (Fig. 1a–e), or in the evening (ZT 12 h) when they are inactive (Fig. 1a–e), and cut into blocks including the POA, BSTM, MBH or PAG (Supplementary Fig. 5). GnIH concentration in the brain blocks including the POA or BSTM were high in the morning and decreased in the evening (Fig. 4a,b). GnIH concentration in other brain blocks showed similar trends (Fig. 4c,d). These results suggested that GnIH synthesized by the action of melatonin at night^28^ is stored in GnIH neuronal fibres (Fig. 3a,b,d,e) in the morning, released during the light hours and depleted in the evening. AA in the brain block including the POA or BSTM was low in the morning and increased in the evening (Fig. 4e,f). The change in AA in the other brain blocks showed similar trends (Fig. 4g,h). E2 concentration in the brain block including the POA was low in the morning and increased in the evening (Fig. 4i), most likely by the action of aromatase (Fig. 4e). E2 concentrations in the other brain blocks showed similar trends (Fig. 4j–l). We also confirmed that E2 concentration is significantly high in the POA compared with the telencephalon, optic tectum, cerebellum and testis in the evening (Supplementary Fig. 6).

### Peripheral E2 and testosterone and corticosterone

We also measured daily changes in E2 and testosterone concentrations in the serum because changes in AA or E2 concentration in the brain may have reflected changes in peripheral E2 or testosterone concentration. However, there was no change in E2 and testosterone concentrations in the serum (Supplementary Fig. 7a,b). There was also no change in corticosterone concentration in the serum (Supplementary Fig. 7c).

### Daily changes in GnIH action and E2 synthesis in the POA

**Figure 5:**
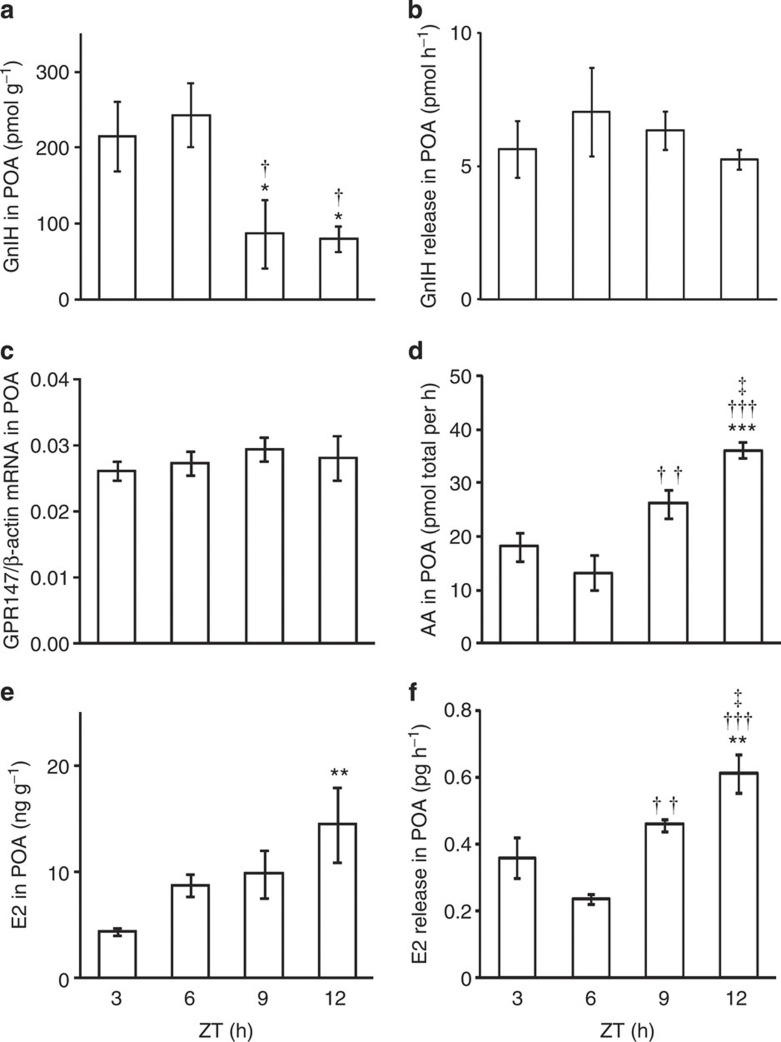
Diurnal changes in GnIH content and release and GnIH receptor mRNA expression and AA and E2 content and release in the POA. (**a**) Diurnal changes in GnIH concentration in the POA. The columns and the vertical lines represent the mean±s.e.m. (*n*=4). DOF=15, F=4.7, *P*=0.022 by one-way ANOVA; *P<0.05 versus ZT 3, ^†^*P*<0.05 versus ZT 6 by Fisher’s PLSD. (**b**) Diurnal changes in GnIH release in the POA. The columns and the vertical lines represent the mean±s.e.m. (*n*=3). (**c**) Diurnal changes in GnIH receptor (GPR147) mRNA expression in the POA. The columns and the vertical lines represent the mean±s.e.m. (*n*=4). (**d**) Diurnal changes in AA in the POA. The columns and the vertical lines represent the mean±s.e.m. (*n*=4). DOF=15, F=14, *P*=0.00029 by one-way ANOVA; ***P<0.001 versus ZT 3, ^††^*P*<0.01, ^†††^*P*<0.001 versus ZT 6, ^‡^, *P*<0.05 versus ZT 9 by Fisher’s PLSD. (**e**) Diurnal changes in E2 concentration in the POA. The columns and the vertical lines represent the mean±s.e.m. (*n*=5). DOF=19, F=3.8, *P*=0.032 by one-way ANOVA; **P<0.01 versus ZT 3 by Fisher’s PLSD. (**f**) Diurnal changes in E2 release in the POA. The columns and the vertical lines represent the mean±s.e.m. (*n*=3). DOF=11, F=14, *P*=0.0016 by one-way ANOVA; **P<0.01 versus ZT 3, ^††^*P*<0.01, ^†††^*P*<0.001 versus ZT 6, ^‡^*P*<0.05 versus ZT 9 by Fisher’s PLSD.

Because changes in GnIH content, AA and E2 content from the morning (ZT 3 h) to the evening (ZT 12 h) were most evident in the POA, we then investigated detailed changes in GnIH content and release, GnIH receptor mRNA expression, AA, E2 content and release in the POA during the light hours (Fig. 5, ZT 3–12 h). GnIH concentration in the POA was high in the morning (ZT 3, 6 h) and decreased in the afternoon (ZT 9 h) and the evening (ZT 12 h) (Fig. 5a). We also measured GnIH release in the POA by using a microdialysis probe inserted in the POA (Supplementary Fig. 5b). GnIH was actively released into the POA throughout the light hours (Fig. 5b).

GPR147 mRNA was also actively synthesized in the POA throughout the light hours (Fig. 5c). AA in the POA was low in the morning (ZT 3, 6 h) and increased in the afternoon (ZT 9 h) and the evening (ZT 12 h) (Fig. 5d).

E2 concentration in the brain block including the POA was low in the morning (ZT 3 h) and gradually increased until the evening (ZT 12 h) (Fig. 5e). E2 collected with GnIH (Fig. 5b) by using a microdialysis probe inserted in the POA (Supplementary Fig. 5b) was low in the morning (ZT 3, 6 h) and increased in the afternoon (ZT 9 h) and the evening (ZT 12 h) (Fig. 5f).

### Stimulation of AA and E2 synthesis in the POA by GnIH

**Figure 6:**
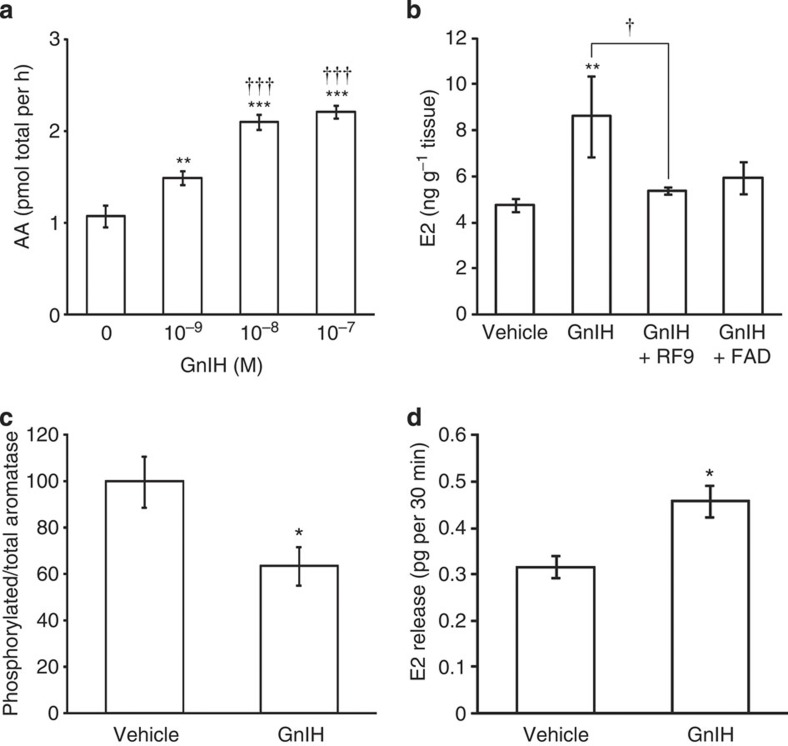
Effect of GnIH administration on AA and E2 content and phosphorylated aromatase and E2 release in the POA. (**a**) AA was measured in the brain blocks including the POA that were incubated with GnIH at various concentrations. The columns and the vertical lines represent the mean±s.e.m. (*n*=6). DOF=23, F=37, *P*=2.6 × 10^−8^ by one-way ANOVA; **P<0.01; ***P<0.001 versus control, ^†††^*P*<0.001 versus 10^−9^ M GnIH by Fisher’s PLSD. (**b**) E2 concentration was measured in the brain blocks including the POA that were incubated with 10^−7^ M GnIH alone (GnIH) or with 10^−6^ M RF9 (GnIH+RF9) or 10^−6^ M fadrozole (GnIH+FAD) or vehicle (Vehicle). The columns and the vertical lines represent the mean±s.e.m. (*n*=6). DOF=23, F=3.2, *P*=0.046 by one-way ANOVA; **P<0.01 versus vehicle. ^†^*P*<0.05 by Fisher’s PLSD. (**c**) Effect of central administration of GnIH on phosphorylated aromatase in the POA. The columns and the vertical lines represent the mean±s.e.m. (*n*=4). DOF=6, *t*=2.6, *P=0.040 by two-tailed Student’s *t*-test. (**d**) Effect of GnIH administration on E2 release in the POA by retromicrodialysis. The columns and the vertical lines represent the mean±s.e.m. (*n*=3). DOF=4, *t*=−3.4, *P=0.027 by two-tailed Student’s *t*-test.

To investigate the function of GnIH in the regulation of AA and E2 synthesis in the POA, we analysed the effect of GnIH administration on AA and E2 synthesis *in vitro* and *in vivo*. GnIH administered to the culture media dose dependently increased the activity of aromatase in the organ cultured brain block including the POA (Fig. 6a). GnIH also increased E2 concentration in the organ cultured brain block including the POA (Fig. 6b). However, concomitant administration of RF9 (refs 29, 30), a GnIH receptor (GPR147) antagonist, or fadrozole^31^,^32^, an aromatase inhibitor, cancelled the stimulatory action of GnIH on E2 synthesis (Fig. 6b). These results suggest that GnIH increases E2 concentration by increasing AA after binding to GPR147 expressed on aromatase cells in the POA.

### GnIH activates AA by dephosphorylation in the POA

It was previously shown that AA in hypothalamic homogenates of male quail is rapidly downregulated by exposure to conditions that enhance phosphorylation, and this inhibition is blocked by kinase inhibitors^33^,^34^,^35^,^36^,^37^. Accordingly, we hypothesized that GnIH may activate aromatase by dephosphorylation of phosphorylated aromatase. We measured phosphorylated aromatase by Phos-Tag SDS-PAGE method^38^ in the brain block including the POA (Supplementary Fig. 5) of birds that were centrally administered with GnIH or vehicle in the morning (ZT 3 h). Central administration of GnIH reduced phosphorylated aromatase in the POA compared with vehicle-administered birds 30 min after administration (Fig. 6c).

If GnIH activates aromatase by dephosphorylation of phosphorylated aromatase in the POA and stimulates the synthesis of E2, GnIH administration in the POA by retrodialysis may increase E2 concentration in the dialysate. GnIH infusion in the POA during the morning (ZT 3–6 h) when natural E2 release in the POA is low (Fig. 5f) increased E2 concentration in the dialysate (Fig. 6d).

### GnIH induces E2 synthesis and inhibits aggressive behaviour

**Figure 7:**
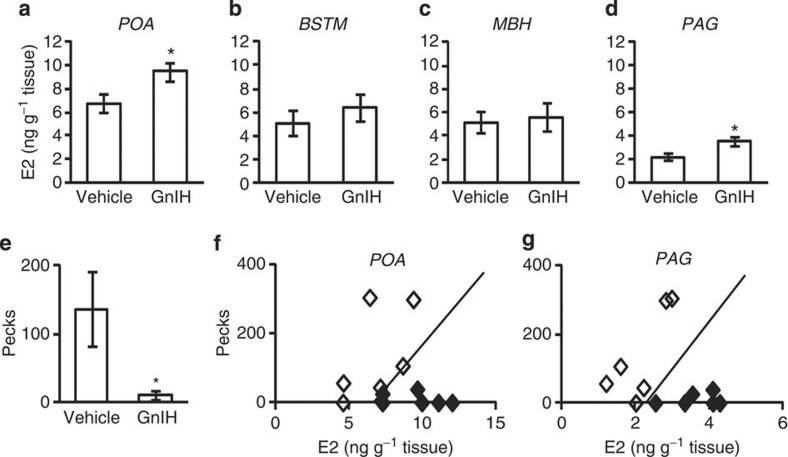
Effect of central administration of GnIH on E2 content in the brain and the frequency of Pecks. (**a**) Effect of GnIH administration on E2 concentration in the POA. The columns and the vertical lines represent the mean±s.e.m. (*n*=6). DOF=10, *t*=−2.4,*P=0.037 by two-tailed Student’s *t*-test. (**b**) Effect of GnIH administration on E2 concentration in the BSTM. The columns and the vertical lines represent the mean±s.e.m. (*n*=6). (**c**) Effect of GnIH administration on E2 concentration in the MBH. The columns and the vertical lines represent the mean±s.e.m. (*n*=6). (**d**) Effect of GnIH administration on E2 concentration in the PAG. The columns and the vertical lines represent the mean±s.e.m. (*n*=6). DOF=10, *t*=−3.0,*P=0.015 by two-tailed Student’s *t*-test. (**e**) Effect of GnIH administration on the number of Pecks in 5 min. The columns and the vertical lines represent the mean±s.e.m. (*n*=6). DOF=10, *t*=2.3,*P=0.047 by two-tailed Student’s *t*-test. (**f**) E2 concentration in the brain blocks including the POA (**a**) was plotted against the number of Pecks (**e**). Open diamonds indicate the results of vehicle administered birds, whereas closed diamonds indicate the results of GnIH administered birds. The line is the discrimination lines by linear discriminant analysis; *P*=0.025. (**g**) E2 concentration in the brain blocks including the PAG (**d**) was plotted against the number of Pecks (**e**). Open diamonds indicate the results of vehicle administered birds, whereas closed diamonds indicate the results of GnIH administered birds. The line is the discrimination lines by linear discriminant analysis; *P*=0.009.

To investigate the physiological role of GnIH action in the stimulation of E2 synthesis in the brain, we analysed the relative effects of central administration of GnIH on E2 concentration in the brain and the frequency of Pecks in individual birds. ICV administration of GnIH increased E2 concentration in the brain blocks including the POA or PAG, 30 min after administration (Fig. 7a,d), associated with a significant decrease in the frequency of Pecks (Fig. 7e) in the morning (ZT 2–4 h), when natural E2 concentration is low (Figs 4i,l and 5e) and the frequency of Pecks is high (Fig. 1b). Linear discriminant analysis showed that ICV administration of GnIH significantly discriminated vehicle-administered groups in terms of the frequency of Pecks (*y*) and E2 concentration (*x*) in the POA (Fig. 7f) or PAG (Fig. 7g). The equations of the discrimination lines were 0.022*y*−1.095*x*+7.315=0 in the POA (*n*=12, *P*=0.025 for *x*, *P*=0.031 for *y*, Fig. 7f) and 0.025*y*−3.491*x*+8.222=0 in the PAG (*n*=12, *P*=0.009 for *x*, *P*=0.027 for *y*, Fig. 7g).

We also measured the effect of GnIH administration on testosterone concentration *in vitro* and *in vivo*. GnIH administration or concomitant administration of RF9 or fadrozole to the culture media did not change testosterone concentration in the brain blocks including the POA (Supplementary Fig. 8a). ICV administration of GnIH did not significantly change testosterone concentration in the brain blocks including the POA, BSTM, MBH or PAG (Supplementary Fig. 8b–e). Linear discriminant analysis did not discriminate GnIH and vehicle-administered groups in terms of the frequency of Pecks (*y*) and testosterone concentration (*x*) in the POA (Supplementary Fig. 8f) or PAG (Supplementary Fig. 8g).

### High E2 concentration inhibits socio-sexual behaviours

**Figure 8:**
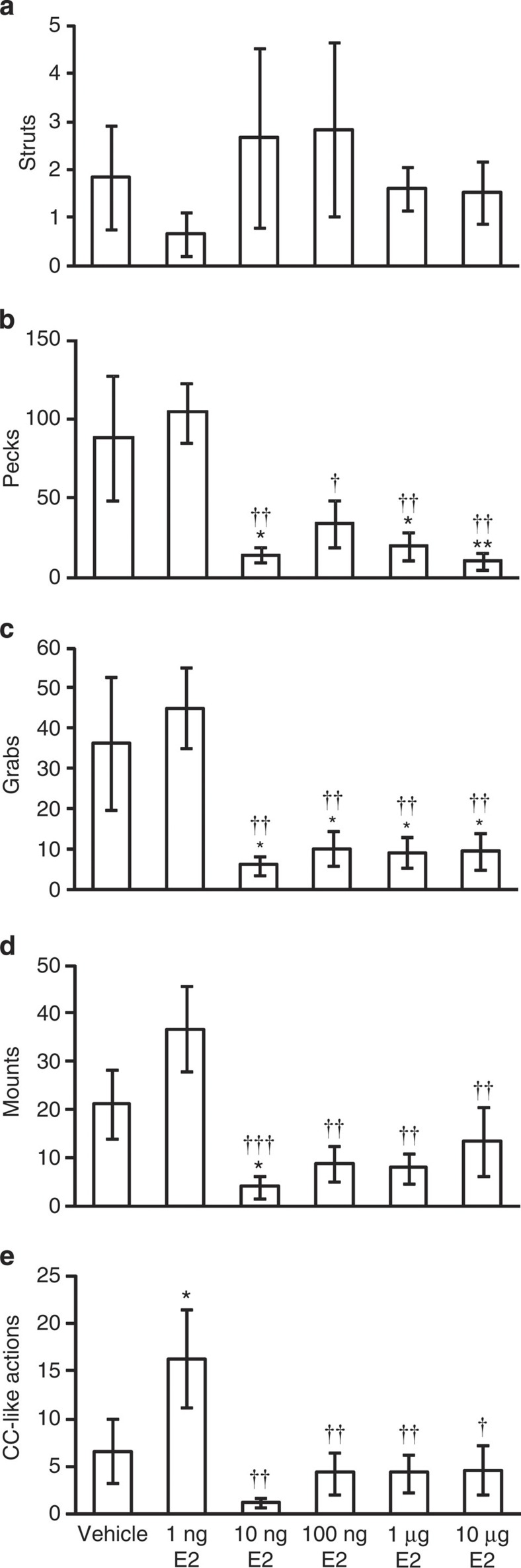
Effect of central administration of E2 on socio-sexual actions of male quail. (**a**) Effect of E2 administration on the number of Struts in 5 min. The columns and the vertical lines represent the mean±s.e.m. (*n*=5). (**b**) Effect of E2 administration on the number of Pecks in 5 min. The columns and the vertical lines represent the mean±s.e.m. (*n*=5). DOF=29, F=4.3, *P*=0.0059 by one-way ANOVA; *P<0.05, **P<0.01 versus vehicle, ^†^*P*<0.05, ^††^*P*<0.01 versus 1 ng E2 by Fisher’s PLSD. (**c**) Effect of E2 administration on the number of Grabs in 5 min. The columns and the vertical lines represent the mean±s.e.m. (*n*=5). DOF=29, F=4.0, *P*=0.0093 by one-way ANOVA; *P<0.05 versus vehicle, ^††^*P*<0.01 versus 1 ng E2 by Fisher’s PLSD. (**d**) Effect of E2 administration on the number of Mounts in 5 min. The columns and the vertical lines represent the mean±s.e.m. (*n*=5). DOF=29, F=4.2, *P*=0.0071 by one-way ANOVA; *P<0.05 versus vehicle, ^††^*P*<0.01, ^†††^*P*<0.001 versus 1 ng E2 by Fisher’s PLSD. (**e**) Effect of E2 administration on the number of CC-like actions in 5 min. The columns and the vertical lines represent the mean±s.e.m. (*n*=5). DOF=29, F=3.1, *P*=0.027 by one-way ANOVA; *P<0.05 versus vehicle, ^†^*P*<0.05, ^††^*P*<0.01 versus 1 ng E2 by Fisher’s PLSD.

The above results indicated that GnIH stimulates E2 synthesis in the brain while inhibiting the frequency of Pecks, suggesting that high concentration of E2 in the brain inhibits male socio-sexual behaviours. We therefore centrally administered various doses of E2 and measured five stereotypic actions of socio-sexual behaviours in the morning (ZT 2–6 h), when their natural expression is high (Fig. 1a–e). Administration of E2 at 1 ng increased the frequency of CC-like action compared with vehicle-administered birds (Fig. 8e). However, ICV administrations of E2 at 10 ng, 100 ng, 1 μg and 10 μg inhibited the frequency of Pecks, Grabs, Mounts and CC-like actions compared with vehicle or 1 ng E2-administered birds (Fig. 8b–e). On the other hand, there was no clear effect of E2 administration on the frequency of Struts (Fig. 8a).

## Discussion

We first measured daily changes in socio-sexual activities of male quail and found that socio-sexual activities were high in the morning and decreased in the afternoon and the evening. Because previous studies have suggested that GnIH decreases socio-sexual activities of male birds^14^,^15^,^16^, we centrally administered GnIH to male quail in the morning when their natural expression of socio-sexual activities was high and showed that GnIH decreases male socio-sexual activities in the morning. We also investigated the effect of GnIH RNAi and concomitant central administration of GnIH on socio-sexual activities of the birds. GnIH RNAi increased the frequency of Struts in the evening when its natural expression was low, and concomitant administration of GnIH inhibited GnIH RNAi induced Struts in the evening. The stimulatory effect of GnIH RNAi and its suppression by GnIH on the activity of quail was more clearly shown when we measured locomotor activity of the birds. On the other hand, there was no effect of GnIH RNAi or GnIH on feeding behaviour of the quail. Because locomotor activity could be regarded as the expression of socio-sexual arousal^14^,^39^,^40^, it is possible that GnIH inhibits socio-sexual arousal of birds resulting in the inhibition of socio-sexual behaviours.

Socio-sexual behaviour of male quail is considered to be androgen dependent because it is reduced by castration and restored by androgen treatment^17^,^18^,^20^. However, there is generally no correlation between the order of aggressiveness of adult male quail and peripheral testosterone concentration^19^,^20^, which was also confirmed in this study. Because it is thought that the action of testosterone on male socio-sexual behaviours requires its aromatization into E2 in the brain (the aromatization hypothesis)^20^,^21^,^22^,^23^,^24^, we initially hypothesized that GnIH might inhibit socio-sexual behaviours of male quail by inhibiting AA and neuroestrogen synthesis. The POA is thought to be the critical site of aromatization and neuroestrogen action for the activation of socio-sexual behaviour of male quail^25^,^26^. We observed abundant GnIH-ir fibres in the vicinity of aromatase cells, and almost all aromatase cells expressed GnIH receptor mRNA in the POA. Abundant GnIH-ir fibres were also observed in the vicinity of aromatase cells in the BSTM, MBH and PAG. These results suggest that GnIH may inhibit socio-sexual behaviours of male quail by regulating the activity of aromatase in the POA and the other brain regions.

We measured daily changes in GnIH content, AA and E2 content in the brain blocks including the POA, BSTM, MBH or PAG. GnIH content was high in the morning and decreased in the evening in the POA. AA and E2 content were low in the morning and increased in the evening in the POA. Other brain regions showed similar trends in the changes in GnIH content, AA and E2 content as the POA. There was no change in serum E2, testosterone and corticosterone concentration between the morning and the evening. To investigate more detailed changes in the activity of GnIH and E2 in the POA, we measured daily changes in GnIH content and release, GPR147 mRNA expression, AA, E2 content and release in the brain blocks including the POA. GnIH was actively released and GPR147 mRNA was highly expressed throughout the day in the POA. Because GnIH is synthesized at night^28^, the decrease in GnIH content in the POA in the evening may be because GnIH stored in its neuronal fibres in the morning was released during the day and depleted in the evening. AA, E2 content and release in the POA were low in the morning and gradually increased until the evening. Because socio-sexual activities of male quail were high in the morning and decreased until the evening, we hypothesized that gradually released GnIH during the day may gradually activate aromatase and stimulate the synthesis of E2 especially in the POA, and higher concentration of neuroestrogen may inhibit male socio-sexual behaviours in the afternoon and the evening.

We accordingly investigated the effect of GnIH administration on AA and E2 synthesis in the POA *in vitro* and *in vivo*. GnIH stimulated the activity of aromatase and increased E2 synthesis in the POA. It is thought that AA is not only controlled in the long term (hours to days) by the transcription of the aromatase gene by steroids but also in the short term (minutes) by phosphorylation by neurotransmitters, such as glutamate^33^. This study adds GnIH as the first neuropeptide that stimulates AA and neuroestrogen synthesis acting in the medium term (minutes to hours). Previous studies have demonstrated that AA is inhibited by phosphorylation in hypothalamic and ovarian homogenates of quail^34^,^35^,^36^ and in various cell lines transfected with human aromatase^37^. The receptor for GnIH is GPR147, which has been shown to couple predominantly through the G_αi_ protein to inhibit cAMP production^41^,^42^,^43^,^44^. We previously investigated the cell signalling process of GPR147 using LβT2 cells, a mouse gonadotrope cell line, and it was demonstrated that mouse GnIH inhibits gonadotropin-releasing hormone (GnRH)-induced gonadotropin subunit gene transcriptions by inhibiting adenylate cyclase/cAMP/PKA-dependent ERK phosphorylation^44^. In this study, the action of GnIH on E2 synthesis in the POA was cancelled by concomitant administration of RF9 (refs 29, 30), a potent GPR147 antagonist, or fadrozole^31^,^32^, an aromatase inhibitor. It was further demonstrated that ICV administration of GnIH reduces phosphorylated aromatase in the POA. Accordingly, it is highly possible that GnIH stimulates neuroestrogen synthesis in the POA by activating aromatase through dephosphorylation after binding to GPR147 expressed on aromatase cells.

To investigate the relationships of GnIH actions in the stimulation of E2 synthesis in the brain and inhibition of socio-sexual activities, we analysed the effect of central administration of GnIH on E2 synthesis and the frequency of Pecks in the same bird. ICV administration of GnIH increased E2 concentration in the brain blocks including the POA or PAG associated with a significant decrease in the frequency of Pecks in the morning. The overall results suggested that GnIH increased E2 synthesis in the brain beyond its optimum concentration for the expression of socio-sexual behaviours. We therefore centrally administered various doses of E2 and measured socio-sexual activities in the morning when their natural expression is high. Central administration of E2 at 1 ng increased the frequency of CC-like action. The same treatment did not stimulate other socio-sexual activities, possibly because their expressions were at their maximum in the morning. ICV administrations of E2 at 10 ng, 100 ng, 1 μg, 10 μg inhibited the number of Pecks, Grabs, Mounts and CC-like actions compared with vehicle- or 1 ng E2-administered birds. On the other hand, all doses of E2 did not significantly stimulate or inhibit the frequency of Struts, one of the actions that were significantly inhibited by GnIH. The link between short-term changes in GnIH, E2 and behaviour may be more complicated than we thought. Further detailed analyses are needed to draw a firm conclusion on the precise physiological functions of GnIH and E2 and its optimum concentration in the activation and inhibition of different socio-sexual activities of male birds.

Even if this study shows that exceedingly high concentration of E2 inhibits the expression of socio-sexual behaviour of male quail, our results are not in conflict with previous studies that studied the function of aromatase or E2 in socio-sexual behaviours of birds, fish and mammals. Pioneering studies in birds that showed the importance of aromatase for sexual behaviour and the stimulatory effect of E2 on sexual behaviour used castrated quail^45^ or reproductively inactivated quail by photoperiodic manuplation^46^, and only single doses of various steroids were administered peripherally for weeks to compare their effects^45^,^46^. Following studies also used castrated or reproductively inactivated quail to test the effect of various steroids that were administered peripherally for days or weeks^20^,^21^,^47^. These studies are likely to have shown the effects of steroids acting in the brain to induce expressions of the genes related to socio-sexual behaviours that were reduced by castration or photoperiodic manipulation.

Cornil *et al.*^48^ showed a rapid effect (15 min) of peripheral E2 injection on the stimulation of copulatory behaviour of male quail^48^. Although the dose of peripherally injected E2 was relatively high (around 50 μg per quail), its concentration may have reduced when it reached the brain to activate this sexual behaviour. E2 was administered to castrated quail pretreated with a dose of testosterone behaviourally ineffective by itself. Accordingly, administered E2 may have also used to initiate gene expression in the brain in their study. Cornil *et al.*^49^ also showed acute effects of vorozole, an aromatase inhibitor, injected peripherally on the inhibition of sexual behaviours of reproductively active male quail. Because it was confirmed that vorozole reached the POA and completely blocked AA in the POA, it is likely that vorozole decreased E2 concentration in the POA below its minimum concentration for the expression of this sexual behaviour, which does not conflict with our results.

Seredynski *et al.*^50^ showed that central administration of E2 at 50–100 μg rapidly increased the expression of sexual motivation quantified by rhythmic cloacal sphincter movements and learned social proximity response but not sexual performance measured by the frequency of cloacal contact movement in castrated male quail that was implanted with testosterone but injected with vorozole. Because our results showed that central administrations of higher doses of E2 at 10 ng to 10 μg inhibited the frequency of CC-like actions in reproductively active male birds, it could be explained that 50–100 μg E2 could not stimulate cloacal contact movement in the study by Seredynski *et al.*^50^, although mechanisms controlling cloacal contact movements against a female bird and CC-like actions against a male bird may be different. We need to perform further detailed experiments to draw a firm conclusion on the role of GnIH and neuroestrogen in the regulation of socio-sexual arousal, motivation and performance in vertebrates^51^,^52^,^53^,^54^,^55^.

## Methods

### Animals

Sexually active male Japanese quail, *Coturnix japonica*, at 2 months of age were obtained from the quail farm (Quail Cosmos, Aichi, Japan) and kept in individual cages (30 cm long, 14 cm wide, 16 cm high). All birds were kept in long day photoperiods (16 h light and 8 h dark, lights on at 0700 hours) and given quail food and tap water *ad libitum*. The experimental protocol was approved in accordance with guidelines prepared by Waseda University (Tokyo, Japan). We followed the ARRIVE guidelines to report this study.

### Measurement of socio-sexual activities

Thirty-two male quail were used to measure daily changes in socio-sexual activity of the birds. Each bird was paired with a stimulus male quail in a cage (35 × 27 × 25 cm) for 5 min during ZT 2.5 and 3.5 h (*n*=8, categorized as ZT 3), ZT 5.5 and 6.5 h (*n*=8, ZT 6), ZT 8.5 and 9.5 h (*n*=8, ZT 9) and ZT 11.5 and 12.5 h (*n*=8, ZT 12). The stimulus male quail was selected 1 day before the experiment, which was sexually mature and physically healthy but subordinate to all other birds. The behaviour of each bird was recorded by using digital video camera and analysed on the computer. We counted the number of Struts, Pecks, Grabs, Mounts and CC-like actions in 5 min (Supplementary Fig. 1). All behavioural measurements were conducted by two observers who were blind to the treatments, and the results were averaged for statistical analyses.

### Effect of GnIH administration on socio-sexual activities

Twenty-four quail were used to test the effect of central administration of GnIH on socio-sexual behaviour of male quail. Brain cannulation to the third ventricle was performed at least 1 week before the behavioural tests according to our established method^14^. In brief, each bird was implanted with a 10 mm 26-gauge stainless steel guide cannula to the third ventricle. The guide cannula was inserted 7 mm ventral from the surface of the skull at the point 3 mm anterior to the lambda and fixed to the skull with dental acrylic under anaesthesia. A 10.5 mm 33-gauge stainless steel obturator was inserted into the guide cannula until the experiment. At the time of infusion, the obturator was removed and a 10 mm 26-gauge stainless steel cannula connected to polyethylene tubing filled with each reagent was inserted into the guide cannula, and the reagent was administered by using a 5 μl microsyringe. Thirty minutes after the administration of GnIH at 100 pmol in 5 μl 0.9% physiological saline (*n*=12) or vehicle (*n*=12), the bird was paired with a stimulus male quail in the behavioural test cage for 5 min during ZT 2 and 4 h. The behavioural test was performed 30 min after administration because previous studies showed that central administrations of the neuropeptides (GnRH and GnIH) were most effective on the behaviour of the birds after 30 min^13^,^56^. Immediately after recording the behaviour of each bird by using digital video camera, each bird was terminated and trunk blood was collected to measure serum testosterone levels by enzyme immunoassay (EIA) (Supplementary Fig. 9).

### Effect of GnIH RNAi and GnIH administration on behaviours

Twenty-four quail were used to test the effect of GnIH RNAi and GnIH administration on locomotor activity, socio-sexual and feeding behaviour (Supplementary Fig. 3c). GnIH siRNA or control RNA at 2 nmol was administered in the third ventricle during ZT 2 and 3 h. The next day, the male birds that had been administered with GnIH siRNA were centrally administered with 100 pmol GnIH in 5 μl sterilized 0.9% physiological saline (*n*=8) or vehicle (*n*=8) in the afternoon (ZT 8–10 h). Male birds that had been administered with control RNA the day before were administered with vehicle (*n*=8). Thirty minutes after the administration of GnIH or vehicle, each male bird was transferred to the behavioural test cage and their locomotor activity was recorded by using a digital camera and the number of times the bird crossed the middle line of the width or the length of the cage was counted in 5 min (Supplementary Fig. 3c). Then the bird was paired with the stimulus male quail in the other behavioural test cage for 5 min to measure its socio-sexual behaviour (Supplementary Fig. 3c). After recording the socio-sexual behaviour of each bird against the stimulus male quail, the bird was transferred back to their individual cages to measure their feeding behaviour. Feeding behaviour was measured by weighing the food that the bird consumed in their individual cages in 3 h (Supplementary Fig. 3c).

### GnIH RNAi

Brain cannulation to the third ventricle was performed according to our established method^14^. To silence GnIH mRNA, 2 nmol of the 25 base-pair double-stranded RNA (sense: 5′-AAGAAGCAUUAAGCCAAGUGCUUAU-3′, antisense: 5′-AUAAGCACUUGGCUUAAUGCUUCUU-3′; Stealth; Invitrogen, Carlsbad, CA, USA) corresponding to *Coturnix japonica* GnIH mRNA sequence (accession code Genbank AB039815, nt 346–370) that does not agree with any other gene in the database was administered in the third ventricle of the experimental bird in the morning (Supplementary Fig. 3c). Control birds were administered with 2 nmol of 25 base-pair double-stranded RNA with a sequence that does not agree with any gene in the database and has the same GC ratio as the GnIH siRNA (sense: 5′-AAGUACGGAAUAACCCGUGUAAUAU-3′, antisense: 5′-AUAUUACACGGGUUAUUCCGUACUU-3′ Stealth Control; Invitrogen) in the morning (Supplementary Fig. 3c). Two nanomoles of GnIH siRNA was estimated to be 1 × 10^5^ times greater than the amount of GnIH mRNA expressed in the brain. GnIH siRNA or control was infused in 5 μl sterilized 0.9% physiological saline.

### Daily changes in GnIH action and E2 synthesis and steroids

Thirty quail were used to measure daily changes in GnIH concentration, AA and E2 concentration in the brain blocks, including the POA, BSTM, MBH or PAG (Supplementary Fig. 5), and E2, testosterone and corticosterone concentration in the serum. Brains were collected during ZT 2.5 and 3.5 h (ZT 3) or ZT 11.5 and 12.5 h (ZT 12), and four brain blocks from each brain were dissected and frozen in liquid nitrogen and stored at −80 °C until assay. The brain blocks collected at the same time (ZT 3 or 12) were used to measure GnIH concentration (*n*=5), AA (*n*=5) and E2 concentration (*n*=5). GnIH, E2, testosterone and corticosterone levels were measured by EIA (Supplementary Fig. 9).

Sixty-eight quail were used to measure daily changes in GnIH concentration, GnIH receptor (GPR147) mRNA expression, AA and E2 concentration in the brain blocks including the POA (Supplementary Fig. 5). Brains were collected during ZT 2.5 and 3.5 h (ZT 3) or ZT 5.5 and 6.5 h (ZT 6) or ZT 8.5 and 9.5 h (ZT 9) or ZT 11.5 and 12.5 (ZT 12) and the brain blocks including the POA were frozen in liquid nitrogen and stored at −80 °C until assay. The brain blocks collected at the same time (ZT 3 or 6 or 9 or 12) were used to measure GnIH concentration (*n*=4), GnIH receptor (GPR147) mRNA expression (*n*=4), AA (*n*=4) and E2 concentration (*n*=5). GnIH receptor mRNA level was measured by real-time quantitative PCR. GnIH and E2 levels were measured by EIA (Supplementary Fig. 9).

Three quail were used to measure daily changes in GnIH and E2 release in the POA by microdialysis. Guide cannulae were attached and dummy cannulae were inserted in the POA (Supplementary Fig. 5b) 1 week before the experiment. One day before the experiment, dummy cannulae were removed, microdialysis probes (PEP-X-0Y, membrane 0.44 mm o.d., 0.34 mm i.d., 1 mm long, 1,000 kDa cut off; Eicom, Kyoto, Japan) were inserted and artificial cerebrospinal fluid (aCSF; 122 mM NaCl, 1.3 mM CaCl_2_, 1.2 mM MgCl_2_, 3.0 mM KH_2_PO_4_, 25.0 mM NaHCO_3_, 0.15% bovine serum albumin; pH 7.4; filtered with 0.2 μm membrane) was perfused at 10 μl min^−1^ for 2 h during ZT 12 and 15 h. Inlet and outlet tubes were removed, but microdialysis probes were kept inserted in the POA during the night. On the day of the experiment, inlet and outlet tubes were connected to the microdialysis probe and aCSF was perfused at 10 μl min^−1^ for 30 min during ZT 0.5 and 1 h and the flow rate was changed to 2 μl min^−1^. Dialysate was collected from ZT 2 until 14 h in collection tubes that were kept on ice. Collection tubes were changed at ZT 5, 8, 11 h so that GnIH and E2 released in the POA from ZT 1.5 to 4.5 h (categorized as ZT 3), ZT 4.5 to 7.5 h (ZT 6), ZT 7.5 to 10.5 h (ZT 9) and ZT 10.5 to 13.5 h (ZT 12) were collected in different tubes. Each sample contained 360 μl of the dialysate. Probes were perfused with methylene green at the end of the experiment and the brain was collected and frozen, and the correct insertion of the probe in the POA was confirmed by sectioning of the brain at 50 μm thickness. GnIH and E2 levels were measured by EIA (Supplementary Fig. 9).

### Effect of GnIH administration on AA in the POA *in vitro*

Twenty-four quail were used to measure the effect of GnIH administration on AA in the brain blocks including the POA *in vitro*. Twenty-four brain blocks including the POA (Supplementary Fig. 5) were sampled during ZT 3 and 4 h in medium 199 at room temperature and preincubated in CO_2_ incubator in a humidified 5% CO_2_ and 80% O_2_ atmosphere at 37 °C for 1.5 h. Each brain block was transferred to each well in a 24-well plate containing 1 × 10^−7^ M GnIH (*n*=6), 1 × 10^−8^ M GnIH (*n*=6), 1 × 10^−9^ M GnIH (*n*=6) or control (*n*=6) in 1 ml medium 199. After incubating for 30 min, 1 × 10^6^ c.p.m. [^3^H]androstenedione was added to each well, and the brain blocks were further incubated for 1 h. AA was measured by counting the radioactivity of [^3^H]E2 converted from [^3^H]androstenedione in the organ-cultured brain blocks.

### Effect of GnIH on E2 and testosterone in the POA *in vitro*

Twenty-four quail were used to measure the effect of GnIH administration on E2 and testosterone concentrations in the brain blocks including the POA *in vitro*. Twenty-four brain blocks including the POA (Supplementary Fig. 5) were sampled during ZT 3 and 4 h in medium 199 supplemented with 10 mM Hepes-NaOH at pH 7.4, 100 U ml^−1^ penicillin and 100 μg ml^−1^ streptomycin at room temperature and preincubated in CO_2_ incubator in a humidified 5% CO_2_ and 80% O_2_ atmosphere at 37 °C for 1.5 h. Each brain block was transferred to each well in a 24-well plate containing 1 × 10^−7^ M GnIH alone (*n*=6) or 1 × 10^−7^ M GnIH with 1 × 10^−5^ M RF9 (*n*=6), or 1 × 10^−7^ M GnIH with 1 × 10^−5^ M fadrozole (*n*=6) or control (*n*=6) in 1 ml medium 199 and further incubated for 1.5 h. Brain blocks were collected and stored at −80 °C until assay for E2 and testosterone concentrations by EIA (Supplementary Fig. 9).

### Effect of GnIH on dephosphorylation of aromatase in the POA

Eight quail were used to measure the effect of central administration of GnIH on dephosphorylation of aromatase in the POA *in vivo*. Brain cannulation to the third ventricle was performed at least 1 week before the experiment according to our established method^14^. Thirty minutes after the administration of GnIH peptide at 100 pmol in 5 μl physiological saline (*n*=4) or vehicle (*n*=4) during ZT 2 and 4 h, each bird was terminated and the brain blocks including the POA (Supplementary Fig. 5) were collected and immediately frozen in liquid nitrogen and stored at −80 °C until assay. Phosphorylated aromatase was measured by using mobility shift detection (Phos-Tag SDS PAGE) method^38^ (Supplementary Fig. 10).

### Effect of GnIH administration on E2 release *in vivo*

Three male quail were used to measure the effect of GnIH administration on E2 release in the POA by retromicrodialysis. Guide and dummy cannulae were inserted in the POA (Supplementary Fig. 5b) 1 week before the experiment. One day before the experiment dummy cannulae were removed, microdialysis probes were inserted and aCSF solution were perfused at 10 μl min^−1^ for 2 h during ZT 12 and 15 h. Inlet and outlet tubes were removed but microdialysis probes were kept inserted in the POA during the night. On the day of the experiment, inlet and outlet tubes were connected to the microdialysis probe and aCSF solution was perfused at 10 μl min^−1^ for 1 h during ZT 3 and 4 h and the flow rate was changed to 4 μl min^−1^. Dialysate was collected for 30 min (control, *n*=3), and aCSF solution containing 1 × 10^−5^ M GnIH (*n*=3) was infused for 30 min and the dialysate was collected (120 μl each) and frozen at −20 °C until assay. E2 concentration in the dialysate was measured by EIA (Supplementary Fig. 9).

### Effect of GnIH on the frequency of Pecks and E2 in the POA

Twelve quail were used to test the effect of GnIH on the frequency of Pecks and E2 concentration in the brain blocks including the POA *in vivo*. Brain cannulation to the third ventricle was performed at least 1 week before the experiment according to our established method^14^. Hundred picomoles of GnIH in 5 μl 0.9% physiological saline (*n*=6) or vehicle (*n*=6) was administered in the third ventricle in the morning (ZT 2–4 h). Thirty minutes after the administration of GnIH or vehicle, each male bird was paired with the stimulus male quail in the behavioural test cage for 5 min, and their behaviour was recorded. The brain blocks including the POA were collected immediately after the behavioural tests and stored at −80 °C until assay. E2 concentration in the brain blocks including the POA was measured by EIA (Supplementary Fig. S9).

### Effect of E2 administration on socio-sexual activities

Thirty quail were used to test the effect of central administration of E2 on the socio-sexual activities of male quail. Brain cannulation to the third ventricle was performed at least 1 week before the experiment^14^. Thirty minutes after the administration of E2 at 1 ng (*n*=5), 10 ng (*n*=5), 100 ng (*n*=5), 1 μg (*n*=5) or 10 μg (*n*=5) in 5 μl physiological saline or vehicle (*n*=5), the bird was paired with a stimulus male quail in a behavioural test cage for 5 min during ZT 2 and 6 h. The behaviour of each bird was recorded by using digital video camera and analysed on the computer.

### GnIH and aromatase immunohistochemistry

Four quail brains were perfusion fixed by 4% paraformaldehyde (PFA) during ZT 3 and 6 h and used for GnIH and aromatase immunohistochemistry. Birds were perfused through the heart with 0.1 M phosphate-buffered saline (PBS), followed by 4% PFA. After post fixation in 4% PFA and saturation with 30% sucrose, the brain was sectioned in the frontal plane at 30 μm. Free-floating sections were rinsed in PBS with 0.3% Triton X-100 (PBS-T). The sections were blocked with 5% normal sheep serum in PBS-T for 2 h at room temperature (RT). The sections were incubated with a purified polyclonal antibody raised in rabbit against quail aromatase at a dilution of 1:1,000 for 24 h at 4 °C. The primary antibody used to label aromatase was a polyclonal affinity-purified antibody raised in rabbit against quail recombinant aromatase (anti-ARO; see Foidart *et al.*^57^ and Corfield *et al.*^58^ for the validation of this antibody). After rinsing, the sections were incubated in Alexa Fluor 488 goat anti-rabbit IgG (Invitrogen, Carlsbad, CA, USA) in PBS-T at a dilution of 1:1,000 for 2 h at RT. After washing, the sections were incubated with prelabelled rabbit antiserum against quail GnIH using a Zenon Alexa Fluor 546 kit (Molecular Probes, Eugene, OR, USA) at a dilution of 1:5,000 for 24 h at 4 °C. The specificity of the GnIH antibody was assessed by adsorption tests of the antibody with 1 × 10^−6^ M synthetic quail GnIH^1^,^7^,^8^. After washing, the sections were mounted with mounting medium and visualized by using a Nikon Ellipse E600 microscope equipped with Y-FL Epi-fluorescence (Nikon, Tokyo, Japan).

### Combination of aromatase immunohistochemistry and *in situ* hybridization for GnIH receptor mRNA

Three male quail brains were isolated and immediately frozen on dry ice during ZT 3 and 6 h and used for *in situ* hybridization for GnIH receptor mRNA combined with aromatase immunohistochemistry. *In situ* hybridization was performed as described previously^59^,^60^ with antisense and sense RNA probes. RNA probes at 906 nt were transcribed from a cDNA amplified by PCR using a forward primer, 5′-GTGACCATTGCCATCATCTG-3′ (identical to nucleotides 568–587; accession code GenBank AB183891), and a reverse primer, 5′-CGTGCCTTTATTGCAGTGAC-3′ (complementary to nucleotides 1454–1473; accession code GenBank AB183891), and quail hypothalamic cDNA. The frozen brain was cut coronal at 20 μm on a cryostat (Leica Jung CM3000; Heidelberg, Germany). The sections were mounted on 3-aminopropyltriethoxysilane-coated slides (Matsunami Glass, Osaka, Japan) and stored at −80 °C until use. The sections were rinsed in PBS three times for 3 min. The slides were deproteinized with 10 mg ml^−1^ proteinase K (Invitrogen) and washed in PBS. The slides were acetylated, then washed in PBS with 1% Triton X-100 (Sigma-Aldrich, St Louis, MO, USA). The sections were incubated at room temperature with hybridization buffer, which contained 50% formamide (Wako Pure Chemicals Industry, Osaka, Japan), 5 × saline sodium citrate (SSC), 1 × Denhart’s solution (Fluka Chemie GmbH, Buchs, Switzerland), 250 μg ml^−1^ yeast RNA (Roche Diagnostics), and 500 μg ml^−1^ DNA (Roche Diagnostics). The sections were then hybridized at 72 °C overnight in a hybridization buffer with RNA probes. The sections were rinsed in 0.2 × SSC for 2 h and then blocked for 2 h in a solution of 0.1 M Tris (pH 7.5) and 0.15 M NaCl with 10% sheep serum. The slides were incubated overnight with alkaline phosphatase-conjugated anti-digoxigenin antibody (catalogue #11082736103, Roche Diagnostics, Basel, Switzerland) and a purified polyclonal antibody raised in rabbit against quail aromatase^57^,^58^ at a dilution of 1:1,000 for 24 h at 4 °C. After washing, the sections were incubated for 2 h at RT in Alexa Fluor 488 goat anti-rabbit IgG (Invitrogen) at a dilution of 1:1,000. After washing, the alkaline phosphatase activity was detected by adding an NBT/BCIP stock solution (Roche Diagnostics). All sections were visualized with a Nikon Ellipse E600 microscope equipped with Y-FL Epi-fluorescence (Nikon).

### Measurement of AA

To assess the activity of aromatase in the quail brain, conversion of [^3^H]androstenedione (PerkinElmer, Yokohama, Japan) to [^3^H]E2 was measured biochemically using brain homogenates or organ cultured quail brain blocks. Biochemical analysis in this study was performed as described previously^61^,^62^. To test the effect of GnIH on AA in the brain *in vivo*, 100 pmol GnIH in 5 μl 0.9% physiological saline or vehicle was administered in the third ventricle in the morning (ZT 2–4 h), and the brain blocks were collected 30 min after administration and stored at −80 °C until assay. The brain blocks were homogenated in 200 μl Medium 199 (Medium 199 supplemented with 10 mM Hepes-NaOH at pH 7.4, 100 U ml^−1^ penicillin and 100 μg ml^−1^ streptomycin; Invitrogen) and 1 × 10^6^ c.p.m. [^3^H]androstenedione (equivalent to 361 pmol) in 50 μl Medium 199 was added (substrate concentration was equivalent to 1444, nM) and incubated for 1 h at 37 °C. To test the effect of GnIH on the AA in the brain *in vitro*, brain blocks were sampled in the morning (ZT 2–4 h) in Medium 199 and preincubated for 1.5 h at 37 °C, 5% CO_2_ and 80% O_2_. Thirty minutes after administration of GnIH at various concentrations, 1 × 10^6^ c.p.m. [^3^H]androstenedione (equivalent to 361 pmol) was added to the media (substrate concentration was equivalent to 361 nM) and incubated for 1 h. Steroids were extracted by 2 ml ethyl acetate and dried. The dried steroids were reconstituted with 800 μl 40% acetonitrile and subjected to HPLC analysis by using reversed-phase column, LiChrospher 100 RP-18 (4.0 × 250 mm; Kanto, Tokyo, Japan). The column was eluted with a 30 min linear gradient of 40–70% acetonitrile at a flow rate of 0.7 ml min^−1^, followed by an isocratic elution with 70% acetonitrile. The eluate was counted in a flow scintillation analyzer (Radiomatic 525 TR; PerkinElmer). Standards of tritiated androstenedione and E2 were also chromatographed to detect their elution positions as a reference. The AA was measured as the area under the curve of tritiated E2 divided by the area under the curve of tritiated androstenedione in the chromatograph of each sample (Supplementary Fig. 11). AA in the homogenate of quail POA brain block was characterized and Eadie–Hofstee plot identified its *K*_m_ and *V*_max_ values to be 13.8 nM and 131 fmol per 1 h incubation of 1 mg tissue, respectively (Supplementary Fig. 12). Thus, the substrate concentrations we used to measure aromatase activities in the tissues (1,444 or 361 nM) were high enough to measure the full activity of aromatase

### E2 enzyme immunoassay

E2 concentration in the brain and serum was measured by using an enzyme immunoassay kit (Estradiol EIA Kit, Cayman Chemical Company, MI, USA) according to the manufacturer’s instruction. The brain blocks were homogenized in the mixture of 800 μl ethyl acetate and 800 μl PBS saline. The homogenate was centrifuged at 16,000 *g* for 30 min at 4 °C. The ethyl acetate portion was collected and dried. The dried material was reconstituted in 160 μl EIA buffer, and 100 μl of the sample was used for EIA at duplicate. Accuracy of E2 measurement in the tissue sample was validated by parallelism of the binding of serial twofold dilutions of the POA sample and E2 standard to the E2 antibody (Supplementary Fig. 9). Serum was directly diluted in the EIA buffer at 50 times dilution. Crossreactivity of the first antibody was 100% to E2, 12% to oestrone, 0.3% to oestriol, and less than 0.01% to aldosterone, 17α-oestradiol, progesterone or testosterone.

### Testosterone enzyme immunoassay

Testosterone concentration in the brain and serum was measured by using an enzyme immunoassay kit (Testosterone EIA Kit, Cayman Chemical Company) according to the manufacturer’s instruction. The brain blocks were homogenized in the mixture of 800 μl ethyl acetate and 800 μl PBS. The homogenate was centrifuged at 16,000 *g* for 30 min at 4 °C. The ethyl acetate portion was collected and dried. The dried material was reconstituted in 160 μl EIA buffer, and 100 μl of the sample was used for EIA at duplicate. Serum was directly diluted in the EIA buffer at 50 times dilution. Crossreactivity of the first antibody was 100% to testosterone, 27.4% to 5α-dihydrotestosterone, 18.9% to 5β-dihydrotestosterone, 3.7% to androstenedione, 2.2% to 11-ketotestosterone, 0.51% to 5-androstenediol, 0.2% to epitestosterone, 0.14% to progesterone, 0.11% to testosterone enanthate, 0.05% to androsterone, 0.04% to androsterone sulphate, 0.03% to testosterone sulphate, 0.02% to dehydroepiandrosterone sulphate and less than 0.01% to E2.

### Corticosterone enzyme immunoassay

Corticosterone concentration in the serum was measured by using an enzyme immunoassay kit (Corticosterone EIA Kit, Cayman Chemical Company) according to the manufacturer’s instruction. Serum was directly diluted in the EIA buffer at 50 times dilution. Crossreactivity of the first antibody was 100% to corticosterone, 0.31% to progesterone, 0.06% to aldosterone, 0.03% to testosterone, 0.02% to pregnenolone, 0.01% to 5α-dihydrotestosterone and less than 0.01% to androstenedione.

### GnIH enzyme immunoassay

The frozen brain blocks were boiled for 7 min and homogenized in 5% acetic acid. The homogenate was centrifuged at 16,000 *g* for 30 min at 4 °C. The supernatant was collected and forced through a disposable C-18 cartridge (Sep-Pak Vac 1cc; Waters, Milford, MA, USA). The retained material was then eluted with 60% methanol. The pooled eluate was concentrated in a vacuum evaporator, passed through disposable Ultrafree-MC centrifugal filter units (Millipore, Billerica, MA, USA) and dried. The dried material was reconstituted in 160 μl EIA buffer, and 100 μl of the sample was used for EIA at duplicate. Dialysate of the microdialysis sample was measured directly. The samples were subjected to competitive EIA by using the antiserum raised in a rabbit against GnIH. In brief, different concentrations of GnIH (1–1,000 pmol ml^−1^) and adjusted tissue extracts or dialysate were added with the antiserum against GnIH (1:1,000 dilution) to each antigen-coated well of a 96-well microplate (multiwell plate for ELISA, H-Type; Sumitomo Bakelite, Tokyo, Japan) and incubated for 1 h at 37 °C. After the reaction with alkaline phosphatase-labelled goat anti-rabbit IgG, immunoreactive products were obtained in a substrate solution of *p*-nitrophenylphosphate, and the absorbance was measured at 415 nm on a microtiter plate reader (MTP-120; Corona Electric, Ibaraki, Japan).

### Real-time quantitative PCR

Total RNA was isolated by the Sepasol extraction method (Sepasol-RNA I Super reagent; Nacalai Tesque, Kyoto, Japan). After DNase-I (TaKaRa, Tokyo, Japan) treatment, total RNA was reverse transcribed using oligo (deoxythymidine) 20 primer (Promega, Madison, WI, USA) and reverse transcriptase (M-MLV Reverse Transcriptase; Invitrogen, Carlsbad, CA, USA). Real-time quantitative PCR was conducted by using the StepOnePlus system (Applied Biosystems, Foster City, CA, USA). β-Actin, a housekeeping gene, was used as an internal standard. The PCR primers used to amplify quail GnIH cDNA fragment were 5′-CTCTAGGCGTGCTCCA-3′ (identical to nucleotides 409–424; accession code GenBank AB039815) and 5′-CATCCCTGGTTCAGATTCCT-3′ (complementary to nucleotides 540–559; accession code GenBank AB039815). The PCR primers used to amplify quail GnIH receptor cDNA fragment were 5′-CACTGATGCTGCTTACAGAC-3′ (identical to nucleotides 959–978; accession code GenBank AB183891) and 5′-CTCATTGAAGTAGCCGTAGA-3′ (complementary to nucleotides 1079–1098; accession code GenBank AB183891). The PCR primers for β-actin were 5′-TTGTGATGGACTCTGGTGATG-3′ (identical to nucleotides 389–409; accession code GenBank AF199488) and 5′-TTCTCTCTCGGCTGTGGTG-3′ (complementary to nucleotides 540–558; accession code GenBank AF199488). The final reaction mixture contained SYBR Green Realtime PCR Master Mix (Toyobo, Osaka, Japan), 1 μM each of forward and reverse primers and 1 μg of cDNA. The PCR condition was 95 °C for 2 min, followed by 40 cycles of 95 °C, 15 s; 60 °C, 15 s; 72 °C, 15 s. An external standard curve was generated by dilutions of the purified target PCR product. To confirm the specificity of the amplification, the PCR products were subjected to a melting curve analysis. The GnIH and GPR147 gene expression was normalized by β-actin gene expression using the StepOnePlus 2.0 software (Applied Biosystems).

### Mobility shift detection of phosphorylated aromatase

After samples had been homogenized in 1 ml lysis buffer containing 10 mM Tris–HCl (pH 7.5), 1% NP-40, 1 mM ethylenediamine tetra-acetic acid (EDTA), a phosphatase inhibitor (PhosSTOP, Roche applied science, Basel, Switzerland), and 1 mM phenylmethanesulfonyl fluoride (PMSF), the lysate was centrifuged at 10,000 *g* for 30 min at 4 °C. The supernatant was then diluted to a protein concentration of 2 mg ml^−1^ with lysis buffer, and equal amounts of protein (5 μg) were loaded onto each lane. Each sample was separated by electrophoresis (CompactPAGE AE-7500, ATTO, Tokyo, Japan) in 8% SDS–polyacrylamide gel to detect total aromatase and 8% SDS–polyacrylamide gel containing a selective phosphate-binding tag molecule, Phos-tag (Wako, Osaka, Japan), at 80 μM and 80 μM MnCl_2_ to separate phosphorylated and dephosphorylated aromatase and transferred to a polyvinylidene difluoride (PVDF) membrane (GE Healthcare, UK) via electrotransfer (CompactBLOT, ATTO).

The membrane was preincubated with a blocking solution (ImmunoBlock, DS Pharma Biomedical, Japan) and then incubated with anti-aromatase rabbit polyclonal antibody (self-produced) at room temperature for 2 h. The membrane was subsequently washed with TBS-T buffer (20 mM Tris–HCl, pH 7.5, 150 mM NaCl, 0.5% Tween-20) and then incubated with a horseradish peroxidase-labelled anti-rabbit immunoglobulin antibody (Bio-Rad Laboratories, Hercules, CA, USA) for 2 h. The antibody was diluted with Can Get Signal Immunoreaction Enhancer Solution (Toyobo, Inc., Osaka, Japan). After intensive wash of the membrane with TBS-T buffer, the immunoreactive bands were visualized using Immobilon Western Chemiluminescent HRP Substrate (Millipore, Billerica, MA, USA) (Supplementary Fig. 10). The intensities of the chemiluminescence of specific aromatase bands were digitized using CoolSaver (ATTO) and quantified. The intensity of the phosphorylated aromatase band was divided by the intensity of the total aromatase band and expressed as the percentage of the average value of the vehicle group. Full gel scans can be found in Supplementary Fig. 13.

### Statistical analysis

All experimental data comparing more than two groups were analysed by one-way ANOVA followed by Fisher’s protected least significant difference (PLSD) test. All experimental data comparing two groups were analysed by two-tailed Student’s *t*-test except diurnal changes in E2 concentration, which was analysed by Mann–Whitney *U*-test. The relationships between testosterone concentration and the frequency of socio-sexual activities were analysed by two-sided Pearson’s correlation test. The effect of ICV administration of GnIH on the relation of E2 or testosterone concentration in the brain blocks including the POA or PAG and the frequency of Pecks was analysed by linear discriminant analysis.

## Author contributions

T.U. and K.T. conceived the project. T.U., S.H., Y.T., M.N, K.I, T.H., N.H. and K.T. performed the experiments and wrote the paper.

## Additional information

**How to cite this article:** Ubuka, T. *et al.* Hypothalamic inhibition of socio-sexual behaviour by increasing neuroestrogen synthesis. *Nat. Commun.* 5:3061 doi: 10.1038/ncomms4061 (2014).

## Supplementary Material

Supplementary Information Supplementary Figures 1-13
