# Supplementary material for: Hypothalamic inhibition of socio-sexual behaviour by increasing neuroestrogen synthesis
Source: Nat Commun. 2014 Jan 16;5:3061. doi: 10.1038/ncomms4061 (PMC3905723; doi:10.1038/ncomms4061)
Supplement: Supplementary Information — Supplementary Figures 1-13 [file ncomms4061-s1.pdf]

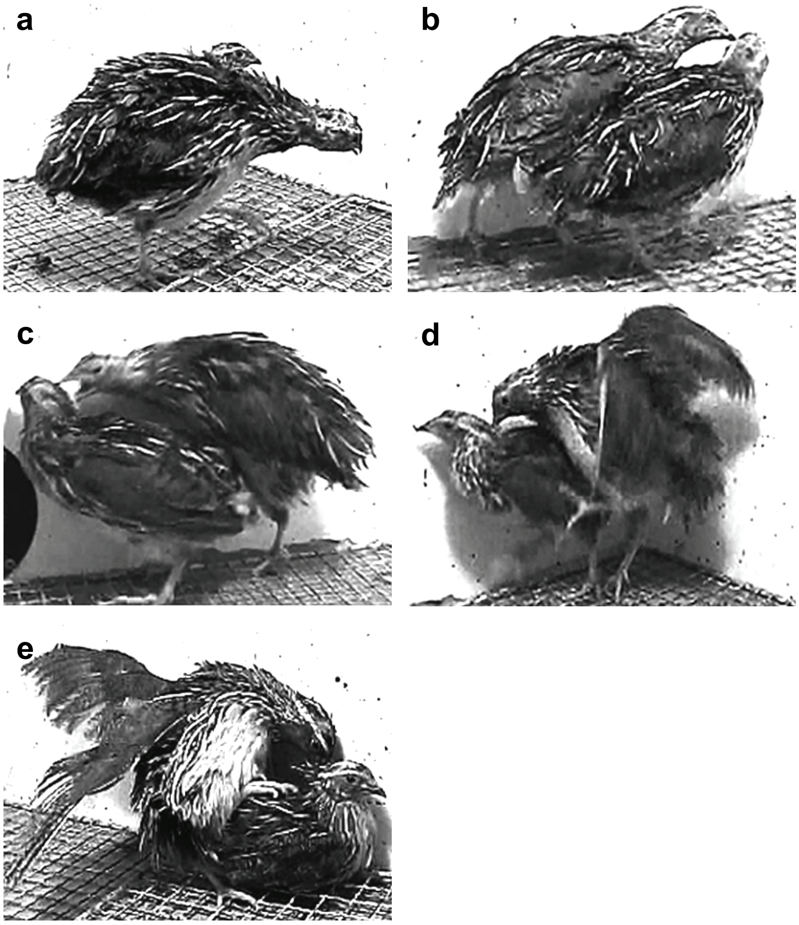

Supplementary Figure S1 | Stereotypic socio-sexual actions when sexually active male quail are paired in a relatively small cage. (**a**) Struts, (**b**) Pecks, (**c**) Grabs, (**d**) Mounts, (**e**) Cloacal Contact (CC)-like actions. When sexually active male quail are paired in a relatively small cage, the dominant male bird displays following stereotypic socio-sexual actions. The dominant male stretches its neck and walks around the subjective male (**a**, Struts). The dominant male approaches the subjective male and chases the opponent if it flees. The dominant male pecks the head of the subjective male (**b**, Pecks). The dominant male grabs the back of the head or neck of the subjective male with its beak (**c**, Grabs). The dominant male mounts on the back of the subjective male and attempts to ride on its back (**d**, Mounts). Finally, the dominant male rides on the back of the subjective male and lowers its cloaca close to the cloaca of the subjective male (**e**, CC-like actions). Mounts and CC-like actions are performed while grabbing the back of the head of the opponent. The behavior of the bird against the stimulus male bird was recorded by a digital camera, and the number of Struts, Pecks, Grabs, Mounts, and CC-like actions were counted in 5 minutes. The stimulus male bird was selected from the stock of sexually active mature birds, which was subjective to all other birds in preliminary behavioral tests. All behavioral measurements were conducted by two observers who were blind to the treatments and the results were averaged for statistical analyses.

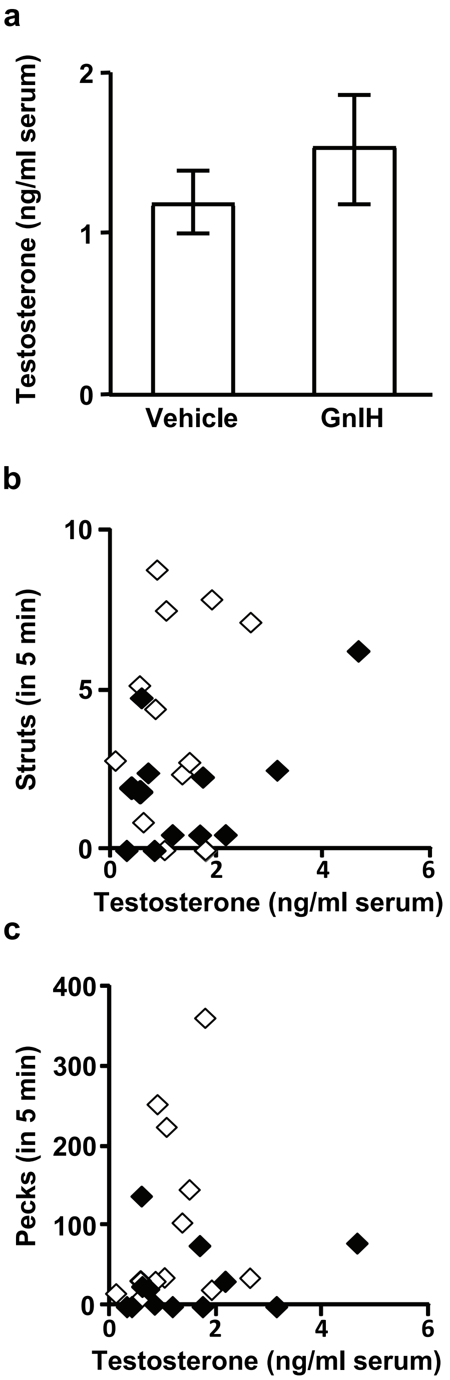

Supplementary Figure S2 | Effect of central administration of GnIH on testosterone concentration in the serum, and relationships between serum testosterone concentration and the number of Struts and Pecks. (**a**) Effect of GnIH administration on testosterone concentration in the serum. The columns and the vertical lines represent the mean ± s.e.m. (n = 12). (**b**) The relationship between serum testosterone concentration and the number of Struts. Open diamonds indicate the results of control birds, whereas closed diamonds indicate the results of experimental birds. n = 24, R = 0.24, P = 0.26 by two-sided Pearson's correlation test. (**c**) The relationship between serum testosterone concentration and the number of Pecks. Open diamonds indicate the results of control birds, whereas closed diamonds indicate the results of experimental birds. n = 24, R = 0.07, P = 0.75 by two-sided Pearson's correlation test.

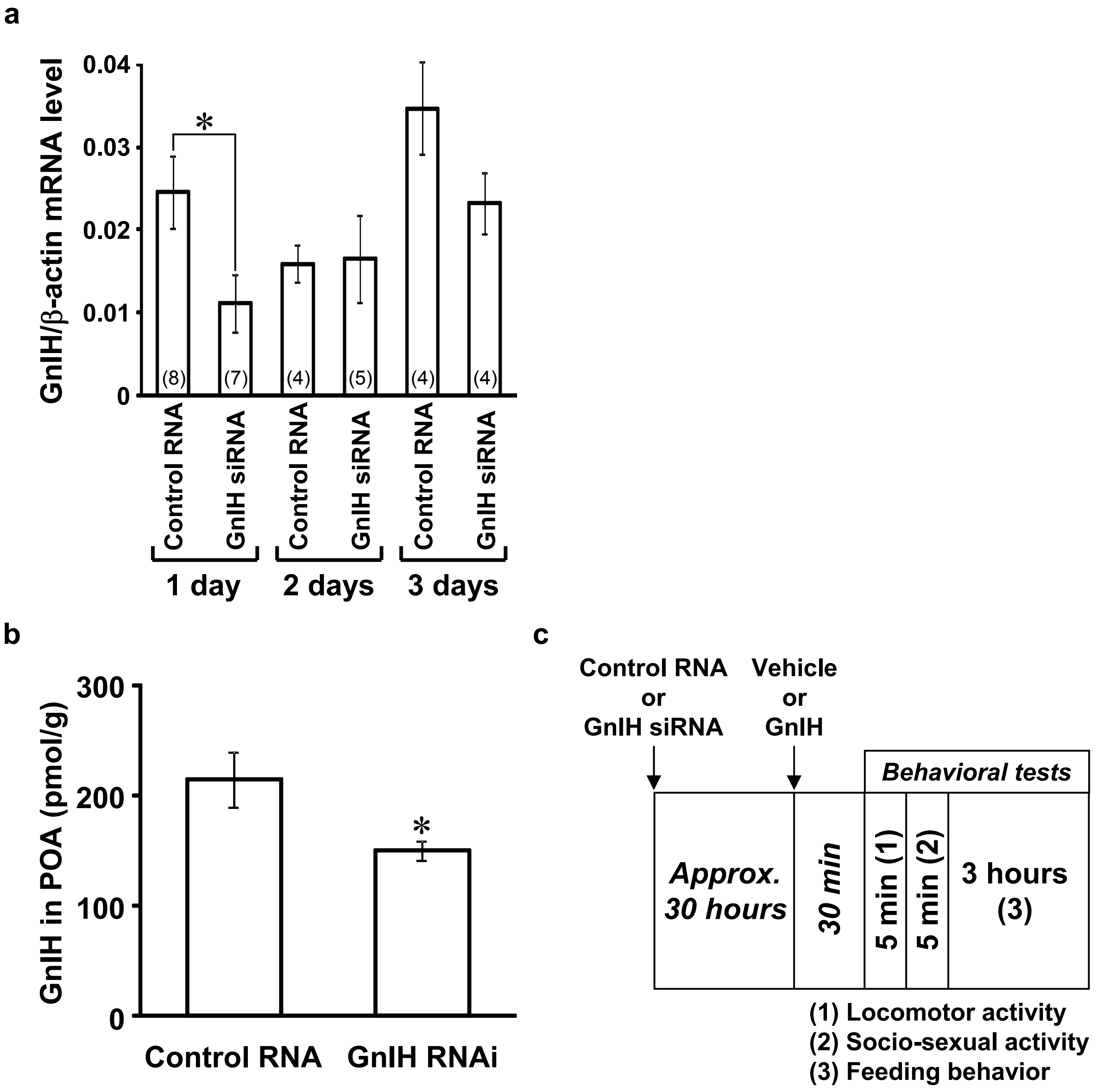

Supplementary Figure S3 | Effect of GnIH RNAi on GnIH mRNA level in the diencephalon and GnIH concentration in the POA, and experimental schedule to test the effect of GnIH RNAi and GnIH administration on locomotor and socio-sexual activity and feeding behavior. (**a**) Effect of GnIH RNAi on GnIH mRNA level in the diencephalon. Control RNA or GnIH siRNA was administered in the third ventricle in the morning (ZT 2-3 h). In the following three days, diencephalons of the birds were collected in the afternoon (ZT 8-10 h) and GnIH mRNA expression relative to β-actin mRNA expression was quantified by RT-real time PCR. The columns and the vertical lines represent the mean ± s.e.m. (number of samples are indicated in the parentheses). Degrees of freedom (DOF) = 13, $t = 2.4$, *, $P = 0.034$ by two-tailed Student's *t*-test. (**b**) Effect of GnIH RNAi on GnIH concentration in the POA. The columns and the vertical lines represent the mean ± s.e.m. ($n = 5$). DOF = 8, $t = 2.5$, *, $P = 0.039$ by two-tailed Student's t-test. (**c**) Experimental schedule to test the effect of GnIH RNAi and GnIH administration on locomotor activity (1), socio-sexual activity (2) and feeding behavior (3).

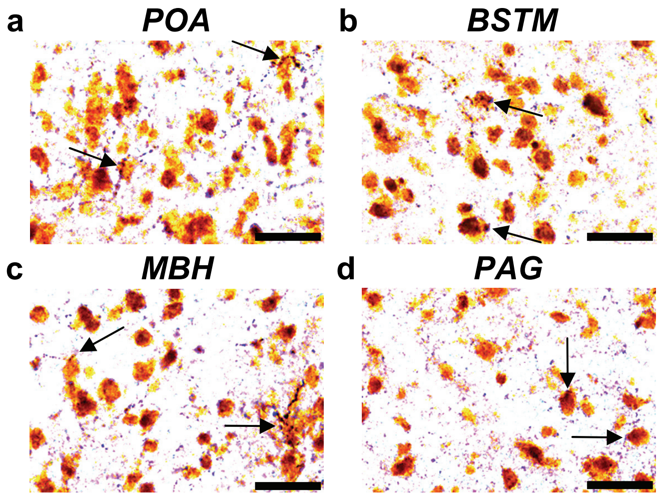

Supplementary Figure S4 | Distribution of GnIH-ir fibers and aromatase-ir cells in the male quail brain. Immunocytochemical analysis of GnIH was conducted with our previous method[9] on the cross sections at 25 µm thickness. The primary antibody used was affinity column purified rabbit anti-quail GnIH antibody. The specificity of the primary antibody was assessed by adsorption tests of the antibody with 1 x 10^{-6} M synthetic quail GnIH. Immunoreactive material was visualized in purple using Vector VIP (Vector Laboratories, Burlingame, CA, USA). After visualization of the location of GnIH, the sections were doubled stained using anti-quail aromatase antibody. The primary antibody used to label aromatase was a polyclonal affinity-purified antibody raised in rabbit against quail recombinant aromatase (anti-ARO; see Foidart *et al.*, 1995[59] and Corfield *et al.*, 2013[60] for the validation of this antibody). Immunoreactive material against aromatase antibody was visualized in brown using diaminobenzidine. The specificity of the anti-ARO was assessed by adsorption tests of the antibody with 1 x 10^{-6} M synthetic quail aromatase. Brain regions and nuclei were identified by Nissl staining of the adjacent sections using a quail brain map[63]. Abundant aromatase-ir cells stained in brown and GnIH-ir fibers stained in purple were observed in the preoptic area (POA, **a**), bed nucleus of the stria terminalis (BSTM, **b**), mediobasal hypothalamus (MBH, **c**), and periaqueductal gray (PAG, **d**). Close appositions of GnIH-ir fibers in the vicinity of aromatase-ir cells are shown by arrows. Bars, 50 µm.

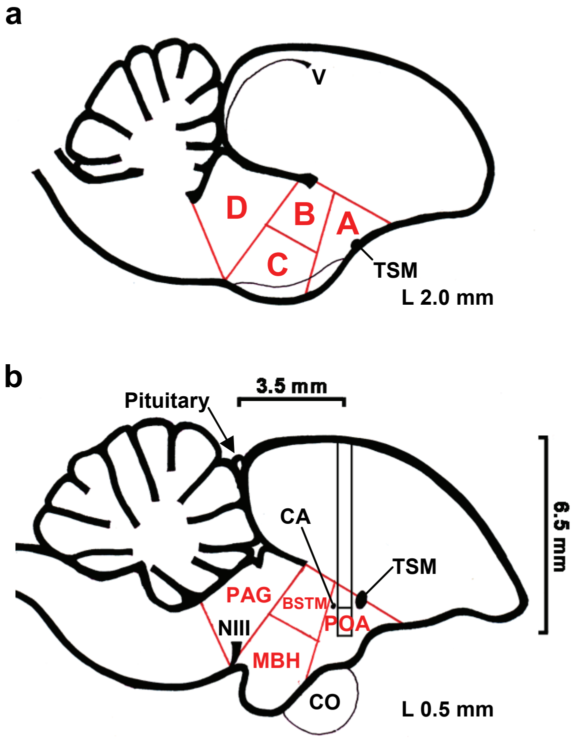

Supplementary Figure S5 | Schematic representation of the sagittal sections of the quail brain. The quail brain was first cut sagittally 2 mm lateral from the midline (L 2.0 mm) to both sides and cut along the red lines shown in (**a**) and (**b**) to separate the brain blocks including the preoptic area (A: POA), bed nucleus of the stria terminalis (B: BSTM), mediobasal hypothalamus (C: MBH), and periaqueductal gray (D: PAG), where aromatase mRNA is distinctively expressed[27]. The microdialysis probe (membrane: 0.44 mm diameter, 1.0 mm long) was inserted 3.5 mm anterior from the pituitary, 0.5 mm lateral from the midline, and 6.5 mm vertical from the surface of the brain as shown in (**b**), according to the quail brain map [Baylé, J. D. *et al.* Stereotaxic topography of the brain of the quail (*Coturnix coturnix japonica*). *J. Physiol. (Paris).* 68, 219–241 (1974)[63]]. V: ventricle, TSM: tractus septomesencephalicus, CA: commissura anterior, NIII: nervus oculomotorius, CO: chiasma opticum.

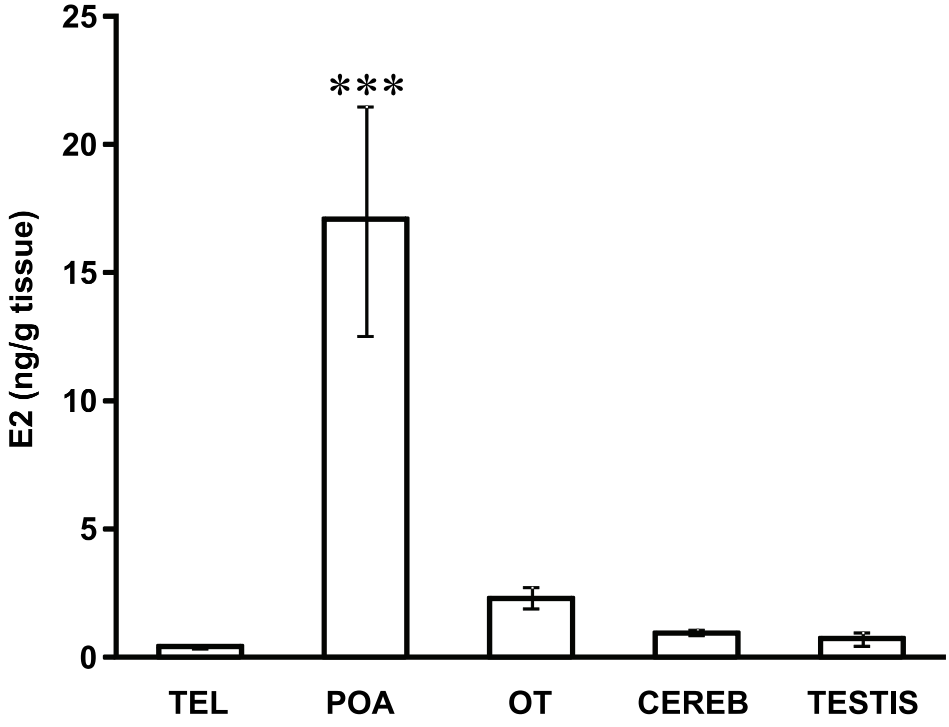

Supplementary Figure S6 | E2 concentration in the telencephlon, the brain block including the POA, optic tectum, cerebellum, and testis in male quail. The tissue was collected during ZT 11.5 and 12.5 h. The brain regions including the preoptic area (POA) was separated according to the brain map depicted in Supplementary Fig. S5. The columns and the vertical lines represent the mean ± s.e.m. (n = 3). Degrees of freedom = 14, F = 13, P = 0.00063 by one-way ANOVA; ***, P < 0.001, POA vs. other tissues by Fisher's PLSD. TEL: telencephalon, OT: optic tectum, CEREB: cerebellum.

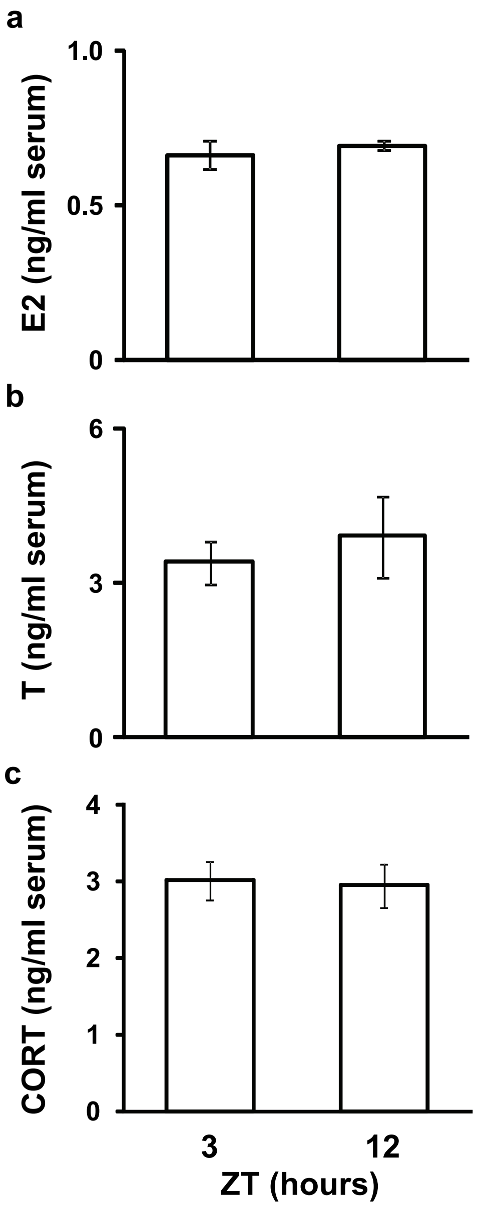

Supplementary Figure S7 | Diurnal changes in E2, testosterone (T) and corticosterone (CORT) concentrations in the sera of male quail. The blood was collected during ZT 2.5 and 3.5 h (ZT 3) or ZT 11.5 and 12.5 h (ZT 12) . The columns and the vertical lines represent the mean ± s.e.m. (*n* = 8).

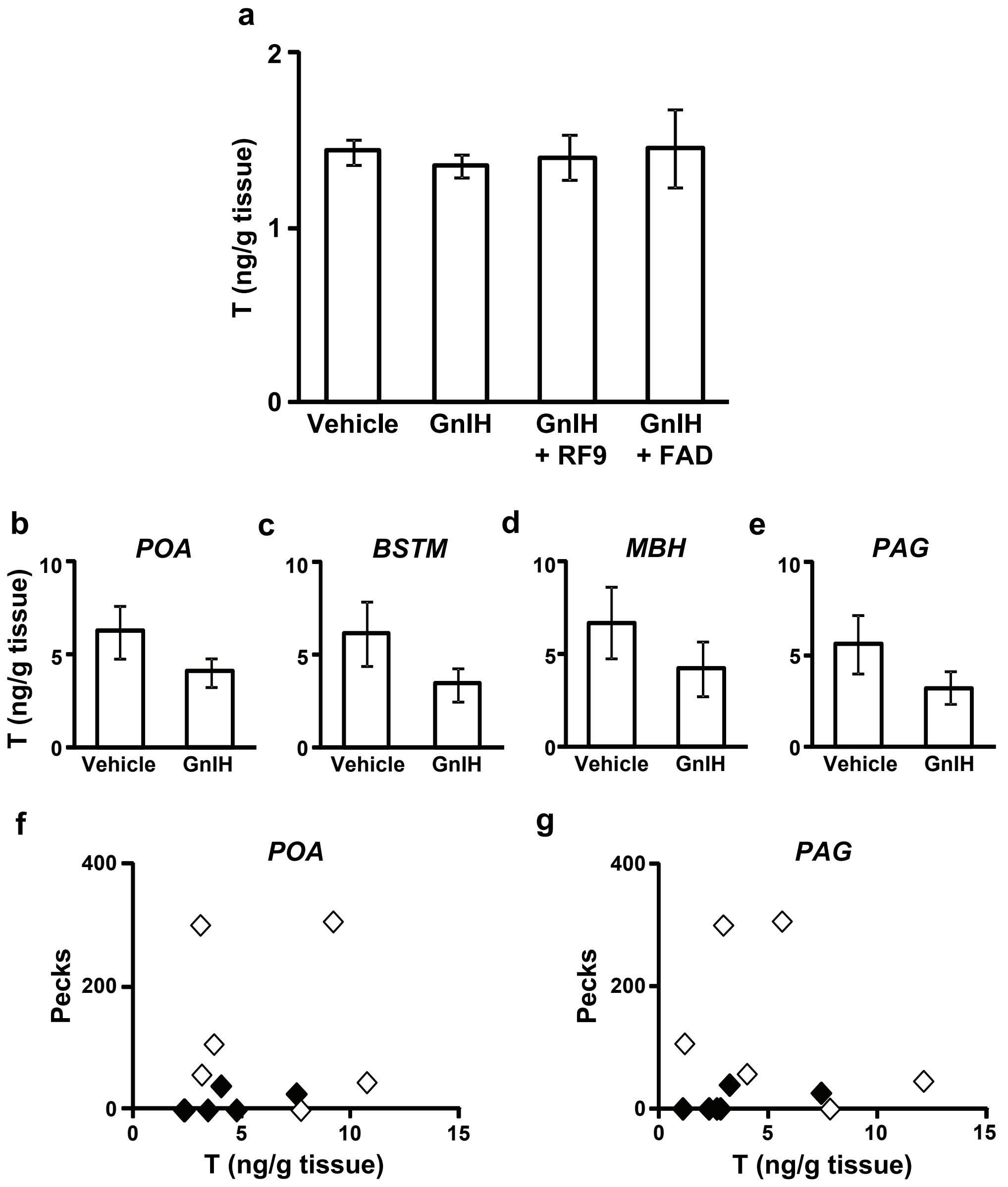

Supplementary Figure S8 | Effect of GnIH administration on testosterone (T) concentration in the quail brain *in vitro* and *in vivo*. (**a**) T concentration in the brain block including the POA incubated with 10^{-7} M GnIH alone or with 10^{-6} M RF9 or 10^{-6} M fadrozole (FAD). The columns and the vertical lines represent the mean ± s.e.m. (*n* = 6). (**b-e**) T concentration in the brain block including POA, BSTM, MBH or PAG that were collected immediately after the 5 minutes' behavioral test performed 30 minutes after central administrations of 100 pmol GnIH or vehicle. The columns and the vertical lines represent the mean ± s.e.m. (*n* = 6). (**f, g**) T concentrations in the brain blocks including the POA (**b**) or PAG (**e**) were plotted against the number of Pecks (Fig. 7e). Open diamonds indicate the results of vehicle administered birds, whereas closed diamonds indicate the results of GnIH administered birds.

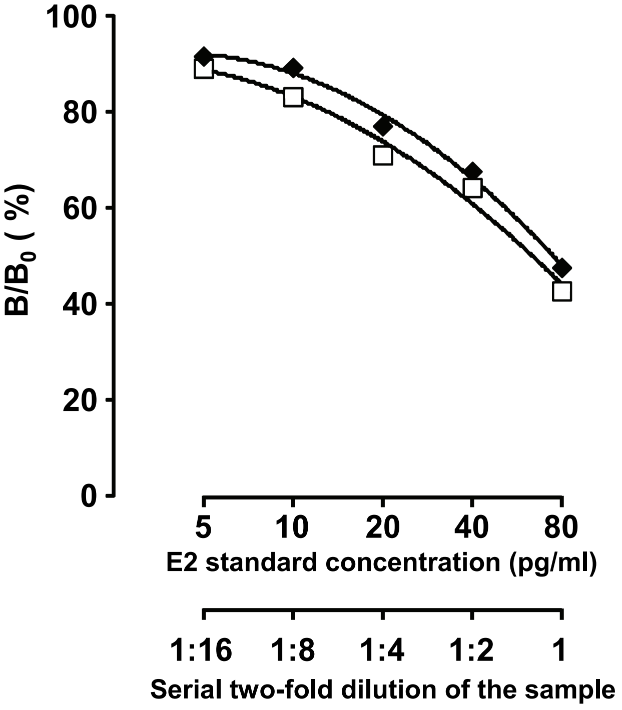

Supplementary Figure S9 | Parallelism of E2 EIA. E2 EIA method was validated by parallelism of the binding of serial two-fold dilutions of the POA sample (open squares) and E2 standard (closed diamond) to the E2 antibody.

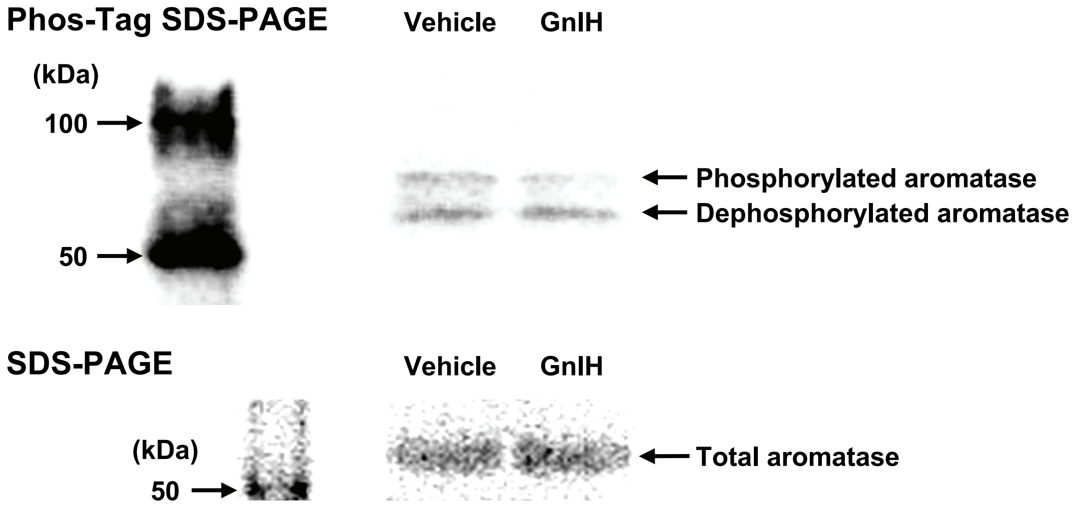

Supplementary Figure S10 | Mobility shift detection of phosphorylated aromatase. Brain blocks containing POA of birds that were centrally administered with 100 pmol GnIH or vehicle were homogenized in lysis buffer containing a phosphatase inhibitor, and the lysate was centrifuged. The supernatant was diluted to a lysis buffer, and equal amounts of protein (5 μg) were separated by electrophoresis in 8% SDS–polyacrylamide gel (SDS-PAGE) to detect total aromatase and 8% SDS–polyacrylamide gel containing a selective phosphate-binding tag molecule (Phos-Tag SDS-PAGE) to separate phosphorylated and dephosphorylated aromatase, and transferred to a PVDF membrane *via* electrotransfer. The membrane was incubated with anti-aromatase antibody and phosphorylated, dephosphorylated, and total aromatase were visualized by chemiluminescence method. This example shows that central administration of GnIH significantly decreased phosphorylated aromatase in the POA. Full gel scan images of Phos-Tag SDS-PAGE and SDS-PAGE are shown in Supplementary Fig. S13.

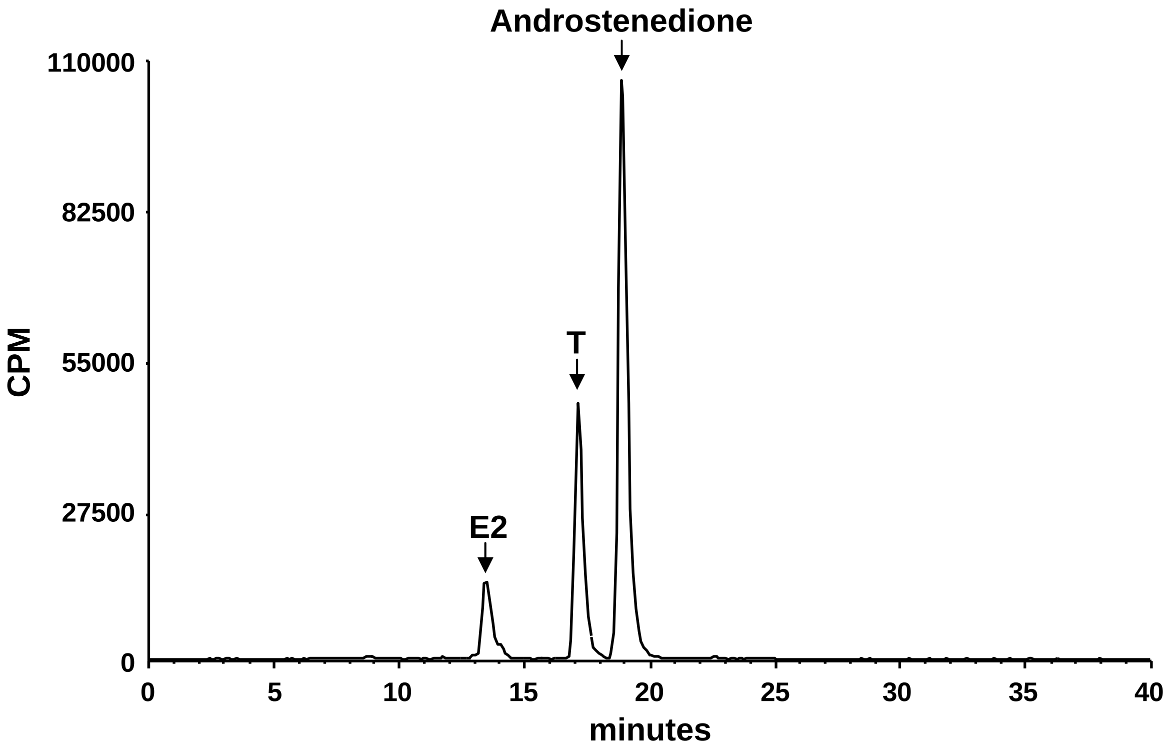

Supplementary Figure S11 | HPLC analysis of E2 formation from androstenedione in the brain blocks including the POA of male quail. The brain block was incubated with [^{3}H]androstenedione for 1 hour and homogenized, and the extract was subjected to HPLC. The arrows indicate the elution positions of the substrate androstendione and its metabolite testosterone (T) and E2.

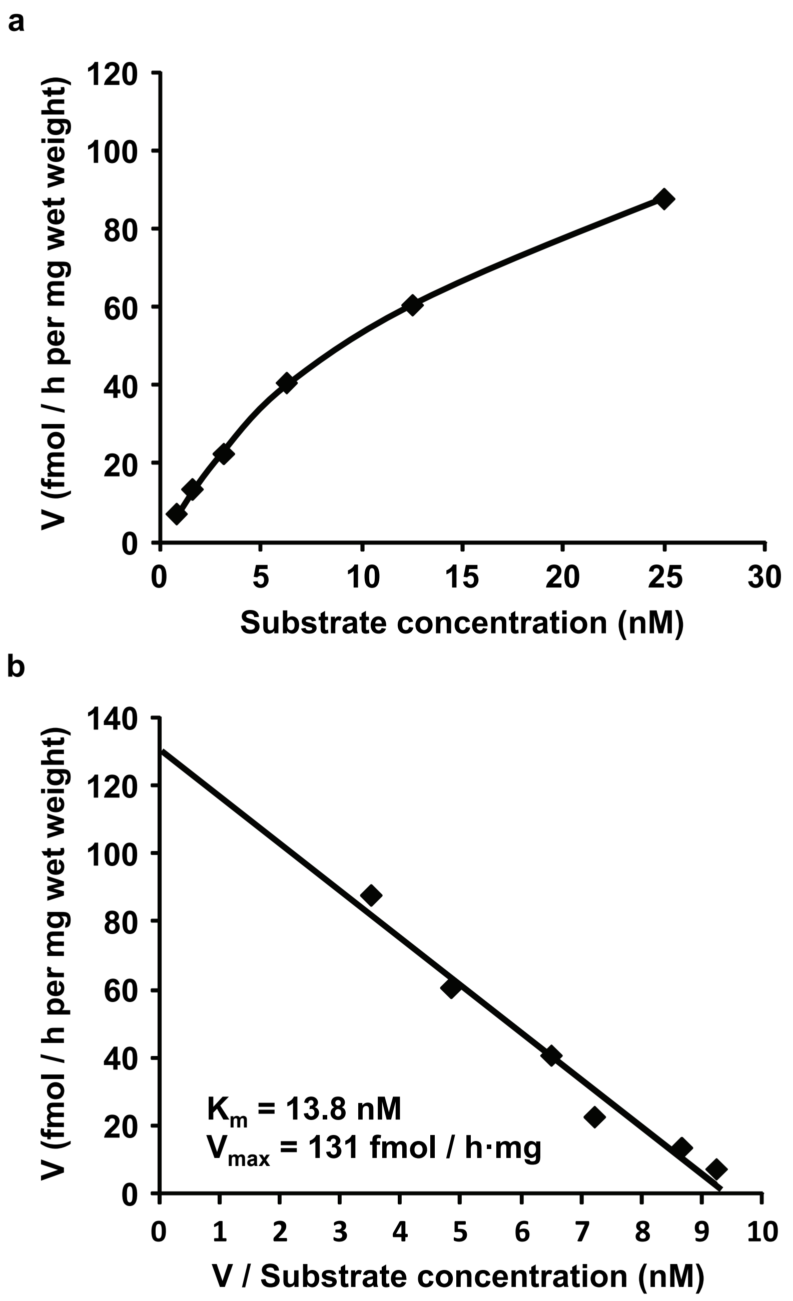

Supplementary Figure S12 | Characterization of aromatase activity in the quail POA brain block. (a) Determination of the activity of aromatase tested by incubating constant amounts of hypothalamic homogenates (1 mg) with increasing concentrations of androstenedione as substrate for 1 hour at 37 ºC. **(b)** Eadie-Hofstee plot of the aromatase activity identified K_m and V_{max} values of the reaction.

Phos-Tag SDS-PAGE

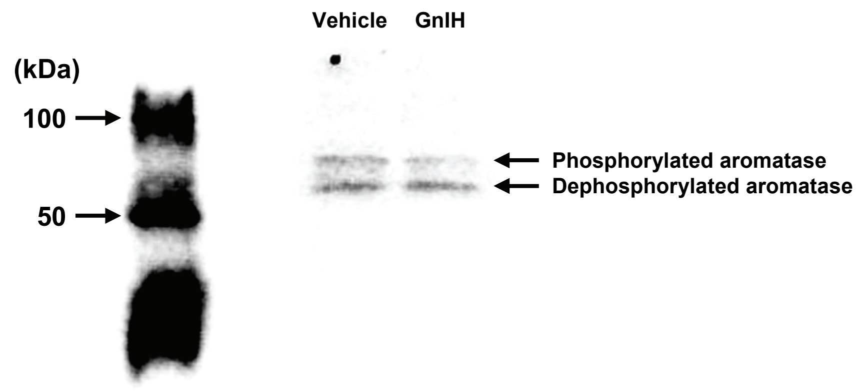

SDS-PAGE

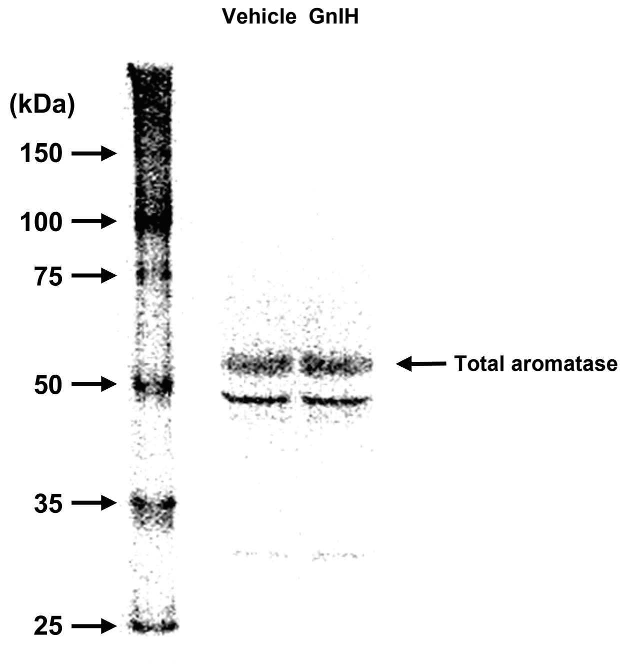

Supplementary Figure S13 | Full gel scan images of Phos-Tag SDS-PAGE and SDS-PAGE. The molecular size marker does not accurately indicate the molecular sizes of the protein in Phos-Tag SDS-PAGE, because of phosphate-binding tag molecule contained in the 8% SDS-polyacrylamide gel and the property of proteins used for molecular size markers. No sample was run on the right lane of the molecular size marker in the Phos-Tag SDS-PAGE to avoid interference with the density of the bands of the samples by molecular size markers. SDS-PAGE shows clear bands of total quail aromatase at the expected size (56 kDa, accession code Genbank AAN04475) as well as some non-specific bands smaller than the 50 kDa molecular size marker.
